# Draft genome of the reindeer (*Rangifer tarandus*)

**DOI:** 10.1093/gigascience/gix102

**Published:** 2017-11-01

**Authors:** Zhipeng Li, Zeshan Lin, Hengxing Ba, Lei Chen, Yongzhi Yang, Kun Wang, Qiang Qiu, Wen Wang, Guangyu Li

**Affiliations:** Jilin Provincial Key Laboratory for Molecular Biology of Special Economic Animals, Institute of Special Animal and Plant Sciences, Chinese Academy of Agricultural Sciences, No. 4899, Juye Street, Jingyue District, Changchun, Jilin province, 130112, P.R. China; Center for Ecological and Environmental Sciences, Northwestern Polytechnical University, No.1 Dongxiang Road, Chang'an District, Xi’an, Shaanxi province, 710129, P.R. China

**Keywords:** *Rangier tarandus*, reindeer, caribou, genomics, whole genome sequencing, assembly, annotation

## Abstract

**Background:**

The reindeer (*Rangifer tarandus*) is the only fully domesticated species in the Cervidae family, and it is the only cervid with a circumpolar distribution. Unlike all other cervids, female reindeer, as well as males, regularly grow cranial appendages (antlers, the defining characteristics of cervids). Moreover, reindeer milk contains more protein and less lactose than bovids’ milk. A high-quality reference genome of this species will assist efforts to elucidate these and other important features in the reindeer.

**Findings:**

We obtained 615 Gb (Gigabase) of usable sequences by filtering the low-quality reads of the raw data generated from the Illumina Hiseq 4000 platform, and a 2.64-Gb final assembly, representing 95.7% of the estimated genome (2.76 Gb according to k-mer analysis), including 92.6% of expected genes according to BUSCO analysis. The contig N50 and scaffold N50 sizes were 89.7 kilo base (kb) and 0.94 mega base (Mb), respectively. We annotated 21 555 protein-coding genes and 1.07 Gb of repetitive sequences by *de novo* and homology-based prediction. Homology-based searches detected 159 rRNA, 547 miRNA, 1339 snRNA, and 863 tRNA sequences in the genome of *R. tarandus*. The divergence time between *R. tarandus* and ancestors of *Bos taurus* and *Capra hircus* is estimated to be about 29.5 million years ago.

**Conclusions:**

Our results provide the first high-quality reference genome for the reindeer and a valuable resource for studying the evolution, domestication, and other unusual characteristics of the reindeer.

## Background Information

**Figure 1:**
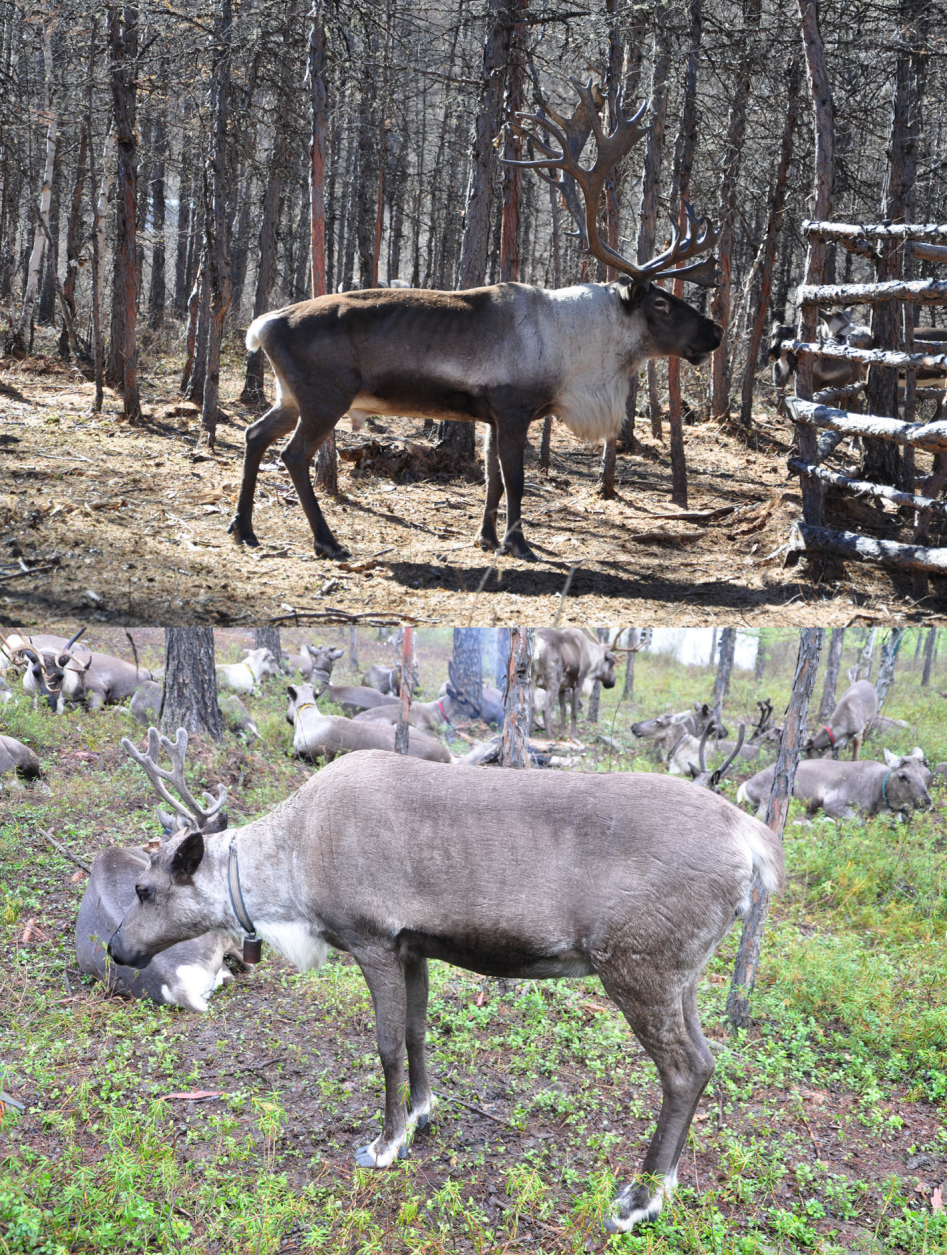
Male (above) and female (below) *Rangier tarandus* individuals, the only cervid species in which both sexes are able to produce velvet antlers. Pictures courtesy of Yifeng Yang from the Institute of Special Animal and Plant Sciences, Chinese Academy of Agricultural Sciences.

The Cervidae is the second largest family in the suborder Ruminantia of the Artiodactyla, which are distributed across much of the globe in diverse habitats, from arctic tundra to tropical forests [1, 2]. Reindeer or caribou (*Rangifer tarandus*, NCBI Taxon ID: 9870) is the only species with a circumpolar distribution (present in boreal, tundra, subarctic, arctic, and mountainous regions of northern Asia, North America, and Europe). It is also the only cervid having been fully domesticated, although some other species have been attempted, such as the sika deer (*Cervus nippon*), which has been semi-domesticated for more than 200 years and still has strong wild nature. Antlers are the defining characteristic of male cervids, belonging to the secondary sexual appendage, which shed and regrow in each year throughout an animal's life. Interestingly, reindeer is the only cervid species in which females regularly grow antlers (Fig. 1). Furthermore, reindeer milk contains a greater amount of proteins and a lower amount of lactose compared with that of bovids [3]. Here, we report a high-quality reindeer reference genome using material from a Chinese individual, which will be useful in elucidating special characteristics of this cervid.

## Data Description

### Animal and sample collecting

Fresh blood was collected from a 2-year-old female reindeer of a domesticated herd maintained by Ewenki (also know as Evenks) hunter-herders in the Greater Khingan Mountains, Inner Mongolia Autonomous Region, China (50.77°N, 121.47°E). The sample was immediately placed in liquid nitrogen, and was then stored at –80°C for later analysis.

### Library construction, sequencing, and filtering

Genomic DNA was extracted from the sample thawed from frozen blood using the DNeasy Blood & Tissue Kit (QIAGEN, Valencia, CA, USA) according to the manufacturer's instructions. Isolated genomic DNA was then used to construct 5 short-insert libraries (200, 250, 350, 400, and 450 bp) and 4 long-insert libraries (3, 6.5, 11.5, and 16 kb) following standard protocols provided by Illumina. Then, 150-bp paired-end sequencing was performed to generate 723.2 Gb of raw data, using a whole genome shotgun sequencing strategy on the Illumina Hiseq 4000 platform (Table S1). To improve the read quality, we trimmed low-quality bases from both sides of the reads and removed reads with more than 5% of uncalled (“N”) bases. Then reads of all libraries were corrected by SOAPec (version 2.03) [4]. Finally, clean reads amounting to 615 Gb were obtained for genome assembly.

### Evaluation of genome size

The estimated genome size is 2.76 Gb according to k-mer analysis, based on the following formula: G = N*(L − 17 + 1)/K_depth (Fig. S1), where N is the total number of reads and K_depth is the frequency of reads occurring more often than others [5]. All the clean reads provide approximately ∼220-fold mean coverage.

### Genome assembly

We used SOAPdenovo (version 2.04; SOAPdenovo2, RRID:SCR_014986) with optimized parameters (pregraph −K 79 −d 0; map -k 79; scaff -L 200) to construct contigs and original scaffolds [5]. All reads were aligned onto contigs for scaffold construction by utilizing the paired-end information. Gaps were filled using reads from 3 libraries (200, 250, and 350 bp) with GapCloser (version 1.12; GapCloser, RRID:SCR_015026) [6]. The final reindeer genome assembly is 2.64 Gb long, including 95.7 Mb (3.6%) of unknown bases, smaller than that of the domestic goat (*Capra hircus*, 2.92 Gb) [7] and similar to that of sheep (*Ovis aries*, 2.61 Gb) [8]. The contig N50 (>200 bp) and scaffold N50 (>500 bp) sizes are 89.7 kb and 0.94 Mb, respectively (Table 1).

**Table 1:** Summary of genome assembly of *Rangier tarandus*

Type	Scaffold (bp)	Contig (bp)
Total number	58 765	117 102
Total length	2 832 785 815	2 732 476 387
N50 length	986 392	91 805
N90 length	151 297	17 480
Max length	4 664 725	770 474
GC content (%)	41.24	40.98

### Quality assessments

We used Benchmarking Universal Single-Copy Orthologs (BUSCO; version 2.0) software to assess the genome completeness (BUSCO, RRID:SCR_015008) [9]. Our assembly covered 92.6% of the core genes, with 3803 genes being complete (Table S2). The feature-response curve (FRC; version 1.3.1) method [10] was then used to evaluate the trade-off between the assembly's contiguity and correctness. The results indicate that it has a similar accumulated curve compared with published high-quality assemblies for other ruminant genomes including cattle, goat, and sheep (Fig. S2). Subsequently, synteny analysis was applied to identify differences between the assembled genome and the domestic goat (*Capra hircus*) genome (Fig. S3); 83.95% of 2 genome sequences could be 1:1 aligned, and the average nuclear distance (percentage of different base pairs in the syntenic regions) was 7.18% (Fig. S4). In addition, the density of different types of break points (edges of structural variation) was about 69.88 per Mb (Table S3). These results suggest that the reindeer genome assembly has of a good level of contiguity and correctness.

### Genome annotation

To annotate the reindeer genome, we initially used LTR_FINDER (LTR_Finder, RRID:SCR_015247) [11] and RepeatModeller (version 1.0.4; RepeatModeler, RRID:SCR_015027) [12] to find repeats. Next, RepeatMasker (version 4.0.5) [13] was used (with -nolow -no_is -norna -parallel 1 parameters) to search for known and novel transposable elements (TE) by mapping sequences against the *de novo* repeat library and Repbase TE library (version 16.02) [14]. Subsequently, tandem repeats were annotated using Tandem Repeat Finder (version 4.07b; with 2 7 7 80 10 50 2000 -d -h parameters) [15]. In addition, we used RepeatProteinMask software [13] with -no LowSimple -*p* value 0.0001 parameters to identify TE-relevant proteins. The combined results indicate that repeat sequences cover about 1.03 Gb, accounting for 39.1% of the reindeer genome assembly (Table S4).

The rest of the reindeer genome assembly was annotated using both *de novo* and homology-based gene prediction approaches. For *de novo* gene prediction, we utilized SNAP (version 2006-07-28), GenScan (GENSCAN, RRID:SCR_012902) [16], glimmerHMM (GlimmerHMM, RRID:SCR_002654), and Augustus (version 2.5.5; Augustus: Gene Prediction, RRID:SCR_008417) [17] to analyze the repeat-masked genome. For homology-based predictions, sequences encoding homologous proteins of *Bos taurus* (Ensemble 87 release), *Ovis aries* (Ensemble 87 release), and *Homo sapiens* (Ensemble 87 release) were aligned to the reindeer genome using TblastN (version 2.2.26; TBLASTN, RRID:SCR_011822) with an (E)-value cutoff of 1 e-5. Genwise (version wise2.2.0) [18] was then used to annotate structures of the genes. The *de novo* and homology gene sets were merged to form a comprehensive, non-redundant gene set using EVidenceModeler software (EVM, version 1.1.1), which resulted in 21 555 protein-coding genes (Table S5). We then compared the reindeer genome with species that were used in homology prediction, and there was no significant difference among the 4 species in gene length and exon length distribution (Fig. S5).

Next, we searched the KEGG, TrEMBL, and SwissProt databases for best matches to the protein sequences yielded by EVM software, using BLASTP (version 2.2.26) with an (E)-value cutoff of 1 e-5, and searched Pfam, PRINTS, ProDom, and SMART databases for known motifs and domains in our sequences using InterProScan software (version 5.18-57.0; InterProScan, RRID:SCR_005829) [19]. At least 1 function was assigned to 19 004 (88.17%) of the detected reindeer genes through these procedures (Table S6). Of them, 14 138 genes were used to do the gene ontology annotation (Fig. S6). The reads from short–insert length libraries then were mapped to the reindeer genome with BWA (version 0.7.12-r1039; BWA, RRID:SCR_010910) [20], then single nucleotide variants (SNVs) were called by SAMtools (version 1.3.1; SAMTOOLS, RRID:SCR_002105) [21]. Finally, we performed SnpEff (version 4.30) [22] to identify the distribution of SNV in the reindeer genome. Finally, a total of 3 353 347 SNVs were found in the genome of the reindeer (Table S7).

In addition, we predicted rRNA-coding sequences based on homology with human rRNAs using BLASTN with default parameters (BLASTN, RRID:SCR_001598). To annotate miRNA and snRNA genes, we searched the Rfam database (release 9.1) with Infernal (version 0.81; Infernal, RRID:SCR_011809) [23] and annotated tRNAs using tRNAscan-SE (version 1.3.1) software with default parameters (tRNAscan-SE, RRID:SCR_010835) [24]. The final results identified 159 rRNAs, 547 miRNAs, 1339 snRNAs, and 863 tRNAs (Table S8).

### Species-specific genes and phylogenetic relationship

**Figure 2:**
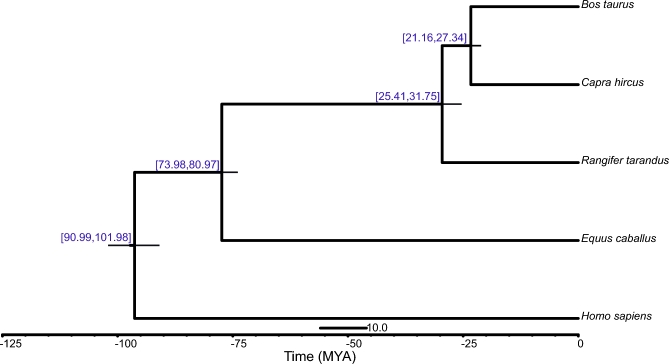
Phylogenetic relationships of *Rangier tarandus* and 4 species based on 4-fold degenerated sites. The blue numbers in the square brackets above the nodes are the 90% confidence intervals of divergence time from the present.

We clustered the detected reindeer genes in families by using OrthoMCL (OrthoMCL DB: Ortholog Groups of Protein Sequences, RRID:SCR_007839) [25] with an (E)-value cutoff of 1 e-5 and a Markov Chain Clustering with default inflation parameter in an all-to-all BLASTP analysis of entries for 5 species (*Homo sapiens, Equus caballus, Capra hircus, Bos taurus*, and *Rangifer tarandus*). The result showed that 335 gene families were specific to the reindeer (Fig. S7). Moreover, we identified 7505 single-copy gene families from these species and aligned coding sequences in the families using PRANK (version 3.8.31) [26]. Subsequently, 4D-sites (4-fold degenerated sites) were extracted to construct a phylogenetic tree by RAxML (version 7.2.8; RAxML, RRID:SCR_006086) [27] with a GTR+G+I model. Finally, phylogenetic analysis using PAML MCMCtree (version 4.5; PAML, RRID:SCR_014932) [28], calibrated with published timings of the divergence of the reference species [29, 30], indicated that *Rangifer tarandus, Bos Taurus*, and *Capra hircus* diverged from a common ancestor approximately 29.5 (25.41-31.75) MYA (Fig. 2). This is consistent with the previous findings from both fossil records and molecular phylogeny analysis [31, 32].

## Conclusion

In summary, we report the first sequencing, assembly, and annotation of the reindeer genome, which will be useful in analysis of the genetic basis of the unique characteristics of reindeer, and broader studies on ruminants.

## Availability of supporting data

The raw sequence data have been deposited in the Short Read Archive (SRA) under accession numbers SRR5763125-SRR5763133. Assemblies, annotations, and other supporting data are also available in the *GigaScience* database, *Giga*DB [33].

## Additional files

Additional file 1:

Supplementary tables_REVISED-1017.doc

Figure S1.pdf

Figure S2.pdf

Figure S3.pdf

Figure S4.pdf

Figure S5.pdf

Figure S6.pdf

Figure S7.pdf

## Abbreviations

bp: base pair; BUSCO: benchmarking universal single-copy orthologs; EVM: EVidenceModeler; FRC: feature-response curves; Gb: giga base; kb:kilo base; Mb: mega base; MYA: million years ago; SNV: single nucleotide variant; TE: transposable element.

## Competing interests

The authors declare that they have no competing interests.

## Author contributions

Z.P.L. collected the samples; Z.S.L., L.C., Z.P.L., Y.Z.Y., K.W., and H.X.B. analyzed the data; Z.S.L., Q.Q., and Z.P.L. wrote the manuscript; W.W. and G.Y.L. conceived the study.

## Supplementary Material

gix102_GIGA-D-17-00152_Original-Submission.pdf

gix102_GIGA-D-17-00152_Revision-1.pdf

gix102_Response-to-Reviewer-Comments_Original-Submission.pdf

gix102_Reviewer-1-Report-(Original-Submission).pdf

gix102_Reviewer-2-Report-(Original-Submission).pdf

Supplement Tables

Supplement Figure S1

Supplement Figure S2

Supplement Figure S3

Supplement Figure S4

Supplement Figure S5

Supplement Figure S6

Supplement Figure S7
