# Supplementary material for: Draft genome of the reindeer (Rangifer tarandus)
Source: Gigascience. 2017 Nov 1;6(12):1–5. doi: 10.1093/gigascience/gix102 (PMC5726476; doi:10.1093/gigascience/gix102)
Supplement: gix102_GIGA-D-17-00152_Revision-1.pdf [file gix102_giga-d-17-00152_revision-1.pdf]

GigaScience

Draft genome of the Reindeer (Rangifer tarandus)

--Manuscript Draft--

**Manuscript Number:**	GIGA-D-17-00152R1
**Full Title:**	Draft genome of the Reindeer (Rangifer tarandus)
**Article Type:**	Data Note
**Funding Information:**	National Natural Science Foundation of China (31501984) — Dr Zhipeng Li<br>Central Public-interest Scientific Institution Basal Research Fund (1610342016026) — Dr Zhipeng Li<br>Talents Team Construction Fund of Northwestern Polytechnical University — Dr Qiang Qiu<br>Talents Team Construction Fund of Northwestern Polytechnical University — Dr Wen Wang
**Abstract:**	Background: Reindeer (Rangifer tarandus) is the only fully domesticated species in the Cervidae family, and is the only cervid with a circumpolar distribution. Unlike all other cervids, female reindeer regularly grow cranial appendages (antlers, the defining characteristics of cervids), as well as males. Moreover, reindeer milk contains more protein and less lactose than bovids' milk. A high quality reference genome of this species will assist efforts to elucidate these and other important features in the reindeer.<br>Findings: We obtained 615 Gb (Gigabase) of usable sequences by filtering the low quality reads of the raw data generated from the Illumina Hiseq 4000 platform, and a 2.64 Gb final assembly, representing 95.7% of the estimated genome (2.76 Gb according to k-mer analysis), including 92.6% of expected genes according to BUSCO analysis. The contig N50 and scaffold N50 sizes were 89.7 kilo base (kb) and 0.94 mega base (Mb), respectively. We annotated 21,555 protein-coding genes and 1.07 Gb of repetitive sequences by de novo and homology-based prediction. Homology-based searches detected 159 rRNA, 547 miRNA, 1,339 snRNA and 863 tRNA sequences in the genome of R. tarandus. The divergence time between R. tarandus, and ancestors of Bos taurus and Capra hircus, is estimated to be about 29.5 million years ago (Mya).<br>Conclusions: Our results provide the first high-quality reference genome for the reindeer, and a valuable resource for studying evolution, domestication and other unusual characteristics of the reindeer.
**Corresponding Author:**	Wen Wang, Ph.D<br><br>CHINA
**Corresponding Author Secondary Information:**	
**Corresponding Author's Institution:**	
**Corresponding Author's Secondary Institution:**	
**First Author:**	Zhipeng Li
**First Author Secondary Information:**	
**Order of Authors:**	Zhipeng Li<br>Zeshan Lin<br>Lei Chen<br>Hengxing Ba<br>Yongzhi Yang

Powered by Editorial Manager® and ProduXion Manager® from Aries Systems Corporation

	Kun Wang
	Wen Wang
	Qiang Qiu
	Guangyu Li
**Order of Authors Secondary Information:**	
**Response to Reviewers:**	We have carefully revised the manuscript according to the reviewers' comments. The point by point responses to the reviewer's comments are listed below, and our responses are in bold and start with dashes "--".<br>Reviewer reports:<br>Reviewer #1<br>In this Data Note the authors describe the first genome assembly for the reindeer. I am impressed by the amount and variety of analyses undertaken to demonstrate the quality of the assembly. That said, I think there are places in the manuscript where the methods could be more fully explained, and broader context given to the results. Specific comments are below<br>--We greatly thank the reviewer to provide us these positive comments to improve the manuscript, especially in the method and result sections.<br><br>Line 20: could be fair to mention that the amount of usable sequence was actually 615 Gb (line 66)<br>--We change this sentence to "We obtained 615 Gb (Gigabase) usable sequences by filtering the low quality reads of the raw data generated from Illumina Hiseq 4000 platform" in lines 21-22.<br><br>Lines 42-45: these two sentences should be re-worded for clarity.<br>--Thanks for your suggestion. In these sentences, we want to address the importance of antlers and the interesting biology of reindeer, which is the only species that females grow antler in the Cervidae. We rewrite these sentences as "Antlers are the defining characteristic of male cervids, belonging to the secondary sexual appendage, which shed and regrow in each year throughout an animal's life. Interestingly, reindeer is the only species that females regularly grow antlers in cervids." in lines 43-46.<br><br>Line 49: replace "special" with "this"<br>-- Changed as suggested.<br><br>Table S1: what is the difference between sequence and physical converge?<br>-- Sequence coverage is the average number of times a base is read, physical coverage is the average number of times a base spanned by paired or mate paired reads. We have added the explanation in the note of the table.<br><br>Lines 69-71: a fuller explanation of the k-mer analysis would be useful. Also, I noted that the distribution in Figure S1 is bimodal. Is this expected? Is it a problem for the analysis? Finally, why not use the traditional c-value estimate of genome size, or at least provide a comparison of the two estimates?<br>-- We have now fully explained the k-mer analysis in lines 70-73 on page 5. The bimodal is common in this kind of analysis. A k-mer is related to an artificial sequence division of K nucleotides extracted iteratively from sequencing reads. We defined the k-mer length as 17 bp; thus, a L bp-long clean sequence would include (L-17 + 1) k-mers. The frequency of each k-mer can be calculated from the genome sequence reads. Typically, k-mer frequencies plotted against the sequence depth gradient follows a Poisson distribution in any given dataset. The k-mer method is regularly used to estimate genome size and heterozogosity in genome projects, and C value method is only used in some less studied taxa with unknown genome size range while the reindeer is one of mammals, whose genome sizes are relatively stable.<br><br>Lines 87-89: it is stated that the accumulation curves in Figure S2 are similar, but to me it looks like the slope for the reindeer is much steeper and more linear than the other genomes. Are they statistically the same? If the reindeer one is different why might that be?<br>-- The horizontal axis represents the error rate and the vertical axis represents the coverage. The error rate of the reindeer is the lowest at the same coverage, indicating

that the high quality assembly of reindeer genome. We have added the explanation in the legend of Figure S2.

Lines 89-96: why was the goat genome chosen for syteny analyses? Is not the cow genome more complete?
-- The goat genome is generated by the third-generation sequencing technology recently with much longer contigs and higher accuracy compared to other ruminants.

Figure S3: please expand the figure legend so that it contains more information as to what is being shown.
-- Thanks for the suggestion. We have improved Figure S3 and added more explanation: The horizontal and vertical axis represents the chromosomes of goat (Capra hircus) and the scaffolds of reindeer (Rangier tarandus), respectively. Those red dots indicate the collinear regions of the two genomes.

Table S4: indicate where % corresponds to % of the genome versus % of elements found.
-- The % in Table S4 indicates that the percentage of repeat regions in reindeer genome. Moreover, we checked the results again and corrected some mistakes which are now marked in yellow.

I would suggest moving the reference to Table S6 from the end of Line 128 to the end of the sentence on Line 127. As it stands now when I went to look at the data I was expecting to see a summary of the functions annotated, not a comparison of how the different software's did. That said, a table summarizing the functions annotated would also be interesting.
-- Thanks for the suggestions. As suggested we have added a Gene ontology annotation to indicate distribution of gene functions in Figure S6.

Lines 130-131: state how many variants were found.
-- We added a sentence in lines 135-136 on page 8 "Finally, a total of 3,353,347 SNVs were found in the genome of reindeer (Table S7)."

Lines 151-153: is this divergence time in line with previous estimates? Please provide citations.
-- The estimated divergence time is consistent with the published results (Ref 1 and 2 listed below). We cited these papers in our manuscript in lines 157-158 of pages 9. "This is consistent with the previous findings from both fossil record and molecular phylogeny analysis (Ref 1 and 2). "
Ref 1.dos Reis M, Inoue J, Hasegawa M, Asher RJ, Donoghue PCJ and Yang Z. Phylogenomic datasets provide both precision and accuracy in estimating the timescale of placental mammal phylogeny. Proceedings of the Royal Society B: Biological Sciences. 2012;279 1742:3491-500.
Ref 2.Bibi F. A multi-calibrated mitochondrial phylogeny of extant Bovidae (Artiodactyla, Ruminantia) and the importance of the fossil record to systematics. BMC Evol Biol. 2013;13 1:166.

Reviewer #2
This is an extremely useful paper to those that are interested in farmed ruminants especially cervids. There are some minor typographical errors which are described below and several minor queries whose answers would improve the text. I checked through the ftp site and the annotation information looks very good and useful. I did not however, download and check each file.
--Thank you very much for your positive comments on this work.

Comments
Major
I am rather confused as nowhere in the text is it described how the assembly scaffolds were ordered and aligned into chromosomes and or genome order. This is important as a number of analyses depend on this aspect. For example the genome comparison with goat (Figure S3V5) and I note the axes of this figure are also cryptically not annotated with either bp or chromosome numbers. I suspect that this did not happen except via homology comparison with another species (sheep or cattle, maybe goat?).

Powered by Editorial Manager® and ProduXion Manager® from Aries Systems Corporation

	Why raise this point? Well to me a high quality assembly actually rests on the scaffolds being ordered and orientated based on data like Hi-C, optical mapping, linkage mapping, LD mapping, or radiation hybrids of which there is no mention. This aspect needs to be clarified and described and commented on.<br>--We are sorry for any unclearness in the description of genome assembly. Indeed, this work didn't assemble the reindeer genome to the chromosomal level, but only to the draft (regular scaffold) level. We actually aligned the reindeer scaffolds to the goat genome which was assembled to the chromosomal level (Ref 3 listed below) to evaluate the quality of our draft assembly. So chromosome information in Figure S3 etc refers to those of goat rather than the reindeer. Usually a high quality draft genome assembly is enough for most biological analyses. If the reindeer chromosomal level information is needed in future studies, one indeed has to use Hi-C, optical mapping, or genetic maps generated with methods like radiation hybrids, and even Hi-C and optical mapping usually can only get longer scaffolds rather than complete chromosomes. We have explained more in the Figure S3 legend avoid ambiguity.<br><br>Ref 3. Bickhart DM, Rosen BD, Koren S, Sayre BL, Hastie AR, Chan S, et al. Single-molecule sequencing and chromatin conformation capture enable de novo reference assembly of the domestic goat genome. Nat Genet. 2017;49 4:643-50.<br><br>Minor * line 19 "species" rather than "specie"<br>-- Corrected as suggested.<br><br>* line 58 The DNA extraction method (and reference) are not described. It is also impossible to call blood frozen and then presumably thawed "fresh blood". This makes me suspect this aspect is unclear to the authors.<br>-- Thank the reviewer to correct this statement. The DNA was extracted from the thawed blood. We rewrite this sentence to "Genomic DNA was extracted from the sample thawed from frozen blood using the DNeasy Blood & Tissue Kit (QIAGEN, Valencia, CA, USA) according to the manufacturer's instructions." in lines 58-60 on page 5.<br><br>* Table s1 needs web address for the deposit numbers.<br>-- We have added the deposit numbers in the Table S1 and their web link.<br><br>* line 153 I suspect figure 1 = figure S7? Figure 1 the precision in the estimated divergence times is excessive and the legend should be altered to make clear it is a range.<br>-- Thank for pointing out this. We have now changed "Figure1" to "Figure S7" in line 157 of page 9. And we used a range rather than a concrete number for the time divergence. We have rewritten the legend of Figure 1 making it clearer.<br><br>* line 168 "he" should be "the".<br>-- Sorry for the typo, we have corrected it.<br><br>* line 172 "libraries" should be "library".<br>-- Corrected as suggested.
**Additional Information:**	
**Question**	**Response**
Are you submitting this manuscript to a special series or article collection?	No
**Experimental design and statistics**<br><br>Full details of the experimental design and statistical methods used should be given in the Methods section, as detailed in our Minimum Standards Reporting Checklist. Information essential to interpreting the data presented should be made available in the figure legends.	Yes

Powered by Editorial Manager® and ProduXion Manager® from Aries Systems Corporation

Have you included all the information requested in your manuscript?	
**Resources**<br><br>A description of all resources used, including antibodies, cell lines, animals and software tools, with enough information to allow them to be uniquely identified, should be included in the Methods section. Authors are strongly encouraged to cite Research Resource Identifiers (RRIDs) for antibodies, model organisms and tools, where possible.<br><br>Have you included the information requested as detailed in our Minimum Standards Reporting Checklist?	Yes
**Availability of data and materials**<br><br>All datasets and code on which the conclusions of the paper rely must be either included in your submission or deposited in publicly available repositories (where available and ethically appropriate), referencing such data using a unique identifier in the references and in the "Availability of Data and Materials" section of your manuscript.<br><br>Have you have met the above requirement as detailed in our Minimum Standards Reporting Checklist?	Yes

Manuscript

Click here to download Manuscript Draft genome of the Reindeer_SCE_ZhipengLI.docx

Draft genome of the Reindeer (*Rangifer tarandus*)

Zhipeng Li[1*], Zeshan Lin[2*], Hengxing Ba[1*], Lei Chen[2], Yongzhi Yang[2], Kun Wang[2], Qiu Qiang[2], Wen Wang[2#],Guangyu Li[1#]

[1] Jilin Provincial Key Laboratory for Molecular Biology of Special Economic Animals, Institute of Special Animal and Plant Sciences, Chinese Academy of Agricultural Sciences, Changchun, 130112, China

[2] Center for Ecological and Environmental Sciences, Northwestern Polytechnical University, Xi'an 710072, China

[*] These authors contributed equally to this work.

[#] Corresponding authors: tcslgy@126.com (GL), wwang@wangwen-lab.org or wwang@mail.kiz.ac.cn (WW)

Contact and ORCID details:

Zhipeng Li, zhplicaas@163.com, ORCID: 0000-0001-8474-045X

Zeshan Lin, linzeshan@126.com, ORCID: 0000-0002-0210-8811

Hengxing Ba, bahengxing@caas.cn, ORCID: 0000-0003-0882-8841

Lei Chen, chenlei@mail.kiz.ac.cn, ORCID: 0000-0003-3989-9239

Yongzhi Yang, yangyongzhi2008@gmail.com, ORCID: 0000-0001-6912-6718

Kun Wang, wk8910@gmail.com, ORCID: 0000-0001-6059-6529

Qiang Qiu, qiuqiang@lzu.edu.cn, ORCID: 0000-0002-9874-271X

WenWang, wwang@mail.kiz.ac.cn, ORCID: 0000-0002-7801-2066

Guangyu Li, tcslgy@126.com, ORCID: 0000-0001-9109-6295

Abstract

Background: Reindeer (*Rangifer tarandus*) is the only fully domesticated species in the Cervidae family, and is the only cervid with a circumpolar distribution. Unlike all other cervids, female reindeer regularly grow cranial appendages (antlers, the defining characteristics of cervids), as well as males. Moreover, reindeer milk contains more protein and less lactose than bovids' milk. A high quality reference genome of this species will assist efforts to elucidate these and other important features in the reindeer.

Findings: We obtained 615 Gb (Gigabase) of usable sequences by filtering the low quality reads of the raw data generated from the Illumina Hiseq 4000 platform, and a 2.64 Gb final assembly, representing 95.7% of the estimated genome (2.76 Gb according to k-mer analysis), including 92.6% of expected genes according to BUSCO analysis. The contig N50 and scaffold N50 sizes were 89.7 kilo base (kb) and 0.94 mega base (Mb), respectively. We annotated 21,555 protein-coding genes and 1.07 Gb of repetitive sequences by *de novo* and homology-based prediction. Homology-based searches detected 159 rRNA, 547 miRNA, 1,339 snRNA and 863 tRNA sequences in the genome of *R. tarandus*. The divergence time between *R. tarandus*, and ancestors of *Bos taurus* and *Capra hircus*, is estimated to be about 29.5 million years ago (Mya).

Conclusions: Our results provide the first high-quality reference genome for the reindeer, and a valuable resource for studying evolution, domestication and other unusual characteristics of the reindeer.

Keywords: *Rangier tarandus*, Reindeer, Caribou, genomics, whole genome sequencing, assembly, annotation

Background information

The Cervidae is the second largest family in the suborder Ruminantia of the Artiodactyla, which are distributed across much of the globe in diverse habitats, from arctic tundra to tropical forests [1, 2]. Reindeer or Caribou (*Rangifer tarandus,* NCBI Taxon ID: 9870) is the only species with a circumpolar distribution (present in boreal, tundra, subarctic, arctic and mountainous regions of northern Asia, North America and Europe). It is also the only cervid having been fully domesticated, although some other species have been attempted, such as the sika deer (*Cervus nippon*), which has been semi-domesticated for more than 200 years and still has strong wild nature. Antlers are the defining characteristic of male cervids, belonging to the secondary sexual appendage, which shed and regrow in each year throughout an animal's life. Interestingly, reindeer is the only cervid species that females regularly grow antlers (**Figure 1**). Furthermore, reindeer milk contains greater amount of proteins, and lower amount of lactose compared to that of bovids [3]. Here, we report a high-quality reindeer reference genome using material from a Chinese individual, which will be useful in elucidating special characteristics of this cervid.

Data description

Animal and sample collecting

Fresh blood was collected from a two-year-old, female reindeer of a domesticated herd maintained by Ewenki (also know as Evenks) hunter-herders in the

Greater Khingan Mountains, Inner Mongolia Autonomous Region, China (50.77° N, 121.47° E). The sample was immediately placed in liquid nitrogen, and was then stored at -80°C for later analysis.

Library construction, sequencing and filtering

Genomic DNA was extracted from the sample thawed from frozen blood using the DNeasy Blood & Tissue Kit (QIAGEN, Valencia, CA, USA) according to the manufacturer's instructions. Isolated genomic DNA was then used to construct five short-insert libraries (200, 250, 350, 400 and 450 base pair, bp) and four long-insert libraries (3, 6.5, 11.5 and 16 kb) following standard protocols provided by Illumina. Then, 150 bp paired-end sequencing was performed to generate 723.2 Gb of raw data, using a whole genome shotgun sequencing strategy on the Illumina Hiseq 4000 platform (**Table S1**). To improve the read quality, we trimmed low-quality bases from both sides of the reads and removed reads with more than 5% of uncalled ("N") bases. Then reads of all libraries were corrected by SOAPec (version 2.03) [4]. Finally, clean reads amounting to 615 Gb were obtained for genome assembly.

Evaluation of genome size

The estimated genome size is 2.76 Gb according to k-mer analysis, based on the following formula: $G = N*(L - 17 + 1) / K\_depth$ (**Figure S1**), where N is the total number of reads, and K_depth is the frequency of reads occurring more often than others [5]. All the clean reads provide approximately ~ 220-fold mean coverage.

Genome assembly

We used SOAPdenovo (SOAPdenovo2 , RRID:SCR_014986)(version 2.04) with optimized parameters (pregraph −K 79 −d 0; map -k 79; scaff -L 200) to construct contigs and original scaffolds [5]. All reads were aligned onto contigs for scaffold construction by utilizing the paired-end information. Gaps were filled using reads from three libraries (200, 250 and 350 bp) with GapCloser (GapCloser, RRID:SCR_015026)(version 1.12) [6]. The final reindeer genome assembly is 2.64 Gb long, including 95.7 Mb (3.6%) of unknown bases, smaller than that of the domestic goat (*Capra hircus*, 2.92 Gb) [7], and similar to that of sheep (*Ovis aries*, 2.61 Gb) [8]. The contig N50 (> 200 bp) and scaffold N50 (> 500 bp) sizes are 89.7 kb and 0.94 Mb, respectively (**Table 1**).

Quality assessments

We used BUSCO (benchmarking universal single-copy orthologs, version 2.0) software to assess the genome completeness (BUSCO , RRID:SCR_015008)[9]. Our assembly covered 92.6% of the core genes, with 3,803 genes being complete (**Table S2**). Feature-response curve (FRC, version 1.3.1) method [10] was then used to evaluate the trade-off between the assembly's contiguity and correctness. The results indicate that it has a similar accumulated curve compared to published high quality assemblies for other ruminant genomes including cattle, goat, and sheep (**Figure S2**). Subsequently, synteny analysis was applied to identify differences between the assembled genome and the domestic goat (*Capra hircus*) genome (**Figure S3**). 83.95%

of two genome sequences could be 1:1 aligned, and the average nuclear distance (percentage of different base pairs in the syntenic regions) was 7.18% (**Figure S4**). In addition, the density of different types of break points (edges of structural variation) are about 69.88 per Mb (**Table S3**). These results suggest that the reindeer genome assembly is of a good level of contiguity and correctness.

Genome annotation

To annotate the reindeer genome we initially used LTR_FINDER (LTR_Finder, RRID:SCR_015247)[11] and RepeatModeller (RepeatModeler, RRID:SCR_015027)(version 1.0.4; http://www.repeatmasker.org/RepeatModeler.html) to find repeats. Next, RepeatMasker (version 4.0.5) [12] was used (with -nolow -no_is -norna -parallel 1 parameters) to search for known and novel transposable elements (TE) by mapping sequences against the *de novo* repeat library and Repbase TE library (version 16.02) [13]. Subsequently, tandem repeats were annotated using Tandem Repeat Finder (version 4.07b; with 2 7 7 80 10 50 2000 -d -h parameters) [14]. In addition, we used RepeatProteinMask software [12] with -no LowSimple -*p* value 0.0001 parameters to identify TE-relevant proteins. The combined results indicate that repeat sequences cover about 1.03 Gb, accounting for 39.1% of the reindeer genome assembly (**Table S4**).

The rest of the reindeer genome assembly was annotated using both *de novo* and homology-based gene prediction approaches. For *de novo* gene prediction, we utilized

SNAP (version 2006-07-28), GenScan (GENSCAN , RRID:SCR_012902)[15], glimmerHMM (GlimmerHMM, RRID:SCR_002654) and Augustus (Augustus: Gene Prediction, RRID:SCR_008417) (version 2.5.5) [16] to analyze the repeat-masked genome. For homology-based predictions, sequences encoding homologous proteins of *Bos taurus* (Ensemble 87 release), *Ovis aries* (Ensemble 87 release) and *Homo sapiens* (Ensemble 87 release), were aligned to the reindeer genome using TblastN (TBLASTN, RRID:SCR_011822)(version 2.2.26) with an (E)-value cutoff of 1 e-5. Genwise (version wise2.2.0) [17] was then used to annotate structures of the genes. The *de novo* and homology gene sets were merged to form a comprehensive, non-redundant gene set using EVidenceModeler software (EVM, version 1.1.1), which resulted in 21,555 protein-coding genes (**Table S5**). We then compared the reindeer genome with species which were used in homology prediction, and there was no significant difference among the four species in gene length and exon length distribution (**Figure S5**).

Next, we searched the KEGG, TrEMBL and SwissProt databases for best matches to the protein sequences yielded by EVM software, using BLASTP (version 2.2.26) with an (E)-value cutoff of 1 e-5, and searched Pfam, PRINTS, ProDom and SMART databases for known motifs and domains in our sequences using InterProScan software (InterProScan , RRID:SCR_005829)(version 5.18-57.0)[18]. At least one function was assigned to 19,004 (88.17%) of the detected reindeer genes through these procedures (**Table S6**). Of them, 14,138 genes were used to do the gene ontology annotation (**Figure S6**). The reads from short-insert length libraries then

were mapped to the reindeer genome with BWA (BWA, RRID:SCR_010910)(version 0.7.12-r1039) [19], then single nucleotide variants (SNVs) were called by SAMtools (SAMTOOLS, RRID:SCR_002105)(version 1.3.1) [20]. Finally, we performed SnpEff (version 4.30) [21] to identify the distribution of SNV in the reindeer genome. Finally, a total of 3,353,347 SNVs were found in the genome of reindeer (**Table S7**).

In addition, we predicted rRNA-coding sequences based on homology with human rRNAs using BLASTN with default parameters (BLASTN, RRID:SCR_001598). To annotate miRNA and snRNA genes we searched the Rfam database (release 9.1) with Infernal (Infernal, RRID:SCR_011809)(version 0.81)[22], and annotated tRNAs using tRNAscan-SE (version 1.3.1) software with default parameters (tRNAscan-SE, RRID:SCR_010835)[23]. The final results identified 159 rRNAs, 547 miRNAs, 1,339 snRNAs and 863 tRNAs (**Table S8**).

Species-specific genes and phylogenetic relationship

We clustered the detected reindeer genes in families by using OrthoMCL (OrthoMCL DB: Ortholog Groups of Protein Sequences, RRID:SCR_007839) [24] with an (E)-value cutoff of 1 e-5, and a Markov Chain Clustering with default inflation parameter in an all-to-all BLASTP analysis of entries for five species (*Homo sapiens, Equus caballus, Capra hircus, Bos taurus,* and *Rangifer tarandus*). The result showed that 335 gene families were specific to the reindeer (**Figure S7**). Moreover, we identified 7,505 single-copy gene families from these species and aligned coding sequences in the families using PRANK (version 3.8.31) [25].

Subsequently, 4D-sites (four-fold degenerated sites) were extracted to construct a phylogenetic tree by RAxML (RAxML, RRID:SCR_006086)(version 7.2.8) [26] with GTR+G+I model. Finally, phylogenetic analysis using PAML MCMCtree (version 4.5) (PAML, RRID:SCR_014932)[27], calibrated with published timings of the divergence of the reference species (http://www.timetree.org/)[28], indicated that *Rangifer tarandus*, *Bos taurus* and *Capra hircus* diverged from a common ancestor approximately 29.5 (25.41-31.75) Mya (**Figure 2**). This is consistent with the previous findings from both fossil record and molecular phylogeny analysis [29, 30].

Conclusion

In summary, we report the first sequencing, assembly and annotation of the reindeer genome, which will be useful in analysis of the genetic basis of the unique characteristics of reindeer, and broader studies on ruminants.

Availability of supporting data

The raw sequence data have been deposited in the Short Read Archive (SRA) under accession numbers SRR5763125-SRR5763133. Assemblies, annotations and other supporting data are also available from the *GigaScience* database[31].

Abbreviations

Gb: giga base; bp: base pair; kb: kilo base; Mb: mega base; TE: transposable element; EVM: EVidenceModeler; BUSCO: benchmarking universal single-copy

orthologs; FRC: feature-response curves; SNV: single nucleotide variant; MYA: million years ago

Acknowledgements

This work was supported by the Natural Science Foundation of China (No. 31501984) and Central Public-interest Scientific Institution Basal Research Fund (No. 1610342016026) to ZPL, and Talents Team Construction Fund of Northwestern Polytechnical University (NWPU) to QQ and WW. Special thanks to Nowbio Biotech Inc., Kunming, China for its remarkable work on DNA library constructions and sequencing.

Competing interests

The authors declare that they have no competing interests.

Authors' contributions

ZPL collected the samples; ZSL, CL ZPL, YZY, KW and HXB analyzed the data; ZSL, QQ and ZPL wrote the manuscript; WW and GYL conceived the study.

References

1. Fernández MH and Vrba ES. A complete estimate of the phylogenetic relationships in ruminantia: a dated species-level supertree of the extant ruminants. Biological Reviews. 2005;80 2:269-302.
2. Hassanin A, Delsuc F, Ropiquet A, Hammer C, Jansen van Vuuren B, Matthee C, et al. Pattern and timing of diversification of Cetartiodactyla (Mammalia, Laurasiatheria), as revealed by a comprehensive analysis of mitochondrial genomes. C R Biol. 2012;335 1:32-50.
3. Young W. Park GFWH. Handbook of milk of non-bovine mammals. Wiley-Blackwell; 2006. ISBN: 978-0-8138-2051-4
4. Luo R, Liu B, Xie Y, Li Z, Huang W, Yuan J, et al. SOAPdenovo2: an empirically improved memory-efficient short-read de novo assembler. GigaScience. 2012;1 1:1-6.
5. Li R, Fan W, Tian G, Zhu H, He L, Cai J, et al. The sequence and de novo assembly of the giant panda genome. Nature. 2010;463 7279:311-7.
6. Li R, Zhu H, Ruan J, Qian W, Fang X, Shi Z, et al. De novo assembly of human genomes with massively parallel short read sequencing. Genome Res. 2010;20 2:265-72.
7. Bickhart DM, Rosen BD, Koren S, Sayre BL, Hastie AR, Chan S, et al. Single-molecule sequencing and chromatin conformation capture enable de novo reference assembly of the domestic goat genome. Nat Genet. 2017;49 4:643-50.
8. Jiang Y, Xie M, Chen W, Talbot R, Maddox JF, Faraut T, et al. The sheep genome illuminates biology of the rumen and lipid metabolism. Science. 2014;344 6188:1168-73.
9. Simão FA, Waterhouse RM, Ioannidis P, Kriventseva EV and Zdobnov EM. BUSCO: assessing genome assembly and annotation completeness with single-copy orthologs. Bioinformatics. 2015;31 19:3210-2.
10. Vezzi F, Narzisi G and Mishra B. Reevaluating assembly evaluations with feature response curves: GAGE and assemblathons. PLoS ONE. 2012;7 12:e52210.
11. Xu Z and Wang H. LTR_FINDER: an efficient tool for the prediction of full-length LTR retrotransposons. Nucleic Acids Res. 2007;35 suppl_2:W265-W8.
12. Tarailo-Graovac M and Chen N. Using RepeatMasker to identify repetitive elements in genomic sequences. Current Protocols in Bioinformatics. John Wiley & Sons, Inc.; 2009.
13. Jurka J, Kapitonov VV, Pavlicek A, Klonowski P, Kohany O and Walichiewicz J. Repbase update, a database of eukaryotic repetitive elements. Cytogenet Genome Res. 2005;110 1-4:462-7.
14. Benson G. Tandem repeats finder: a program to analyze DNA sequences. Nucleic Acids Res. 1999;27 2:573-80.

15. Burge C and Karlin S. Prediction of complete gene structures in human genomic DNA1. J Mol Biol. 1997;268 1:78-94.
16. Stanke M, Keller O, Gunduz I, Hayes A, Waack S and Morgenstern B. AUGUSTUS: ab initio prediction of alternative transcripts. Nucleic Acids Res. 2006;34 suppl_2:W435-W9.
17. Birney E, Clamp M and Durbin R. GeneWise and Genomewise. Genome Res. 2004;14 5:988-95.
18. Philip Jones, David Binns, Hsin-Yu Chang, Matthew Fraser, Weizhong Li, Craig McAnulla, Hamish McWilliam, John Maslen, Alex Mitchell, Gift Nuka, Sebastien Pesseat, Antony F. Quinn, Amaia Sangrador-Vegas, Maxim Scheremetjew, Siew-Yit Yong, Rodrigo Lopez, and Sarah Hunter (2014). InterProScan 5: genome-scale protein function classification. Bioinformatics, Jan 2014; doi:10.1093/bioinformatics/btu031
19. Heng L. Aligning sequence reads, clone sequences and assembly contigs with BWA-MEM. arXiv. 2013;1303.3997
20. Li H, Handsaker B, Wysoker A, Fennell T, Ruan J, Homer N, et al. The sequence alignment/map format and SAMtools. Bioinformatics. 2009;25 16:2078-9.
21. Cingolani P, Platts A, Wang LL, Coon M, Nguyen T, Wang L, et al. A program for annotating and predicting the effects of single nucleotide polymorphisms, SnpEff: SNPs in the genome of *Drosophila melanogaster* strain w (1118); iso-2; iso-3. Fly (Austin). 2012;6 2:80-92.
22. E. P. Nawrocki and S. R. Eddy, Infernal 1.1: 100-fold faster RNA homology searches, Bioinformatics 29:2933-2935 (2013).
23. Lowe TM, Chan PP. tRNAscan-SE On-line: integrating search and context for analysis of transfer RNA genes. Nucleic Acids Res. 2016 Jul 8;44(W1):W54-7. doi: 10.1093/nar/gkw413.
24. Li L, Stoeckert CJ and Roos DS. OrthoMCL: Identification of ortholog groups for eukaryotic genomes. Genome Res. 2003;13 9:2178-89.
25. Löytynoja A and Goldman N. An algorithm for progressive multiple alignment of sequences with insertions. Proc Natl Acad Sci U S A. 2005;102 30:10557-62.
26. Stamatakis A. RAxML version 8: a tool for phylogenetic analysis and post-analysis of large phylogenies. Bioinformatics. 2014;30 9:1312-3.
27. Yang Z. PAML 4: Phylogenetic analysis by maximum likelihood. Mol Biol Evol. 2007;24 8:1586-91.
28. Hedges SB, Marin J, Suleski M, Paymer M, Kumar S. Tree of life reveals clock-like speciation and diversification. Mol Biol Evol. 2015 Apr;32(4):835-45. doi: 10.1093/molbev/msv037.
29. dos Reis M, Inoue J, Hasegawa M, Asher RJ, Donoghue PCJ and Yang Z. Phylogenomic datasets provide both precision and accuracy in estimating the timescale of placental mammal phylogeny. Proceedings of the Royal Society B: Biological Sciences. 2012;279 1742:3491-500.

30. Bibi F. A multi-calibrated mitochondrial phylogeny of extant Bovidae (Artiodactyla, Ruminantia) and the importance of the fossil record to systematics. BMC Evol Biol. 2013;13 1:166.29.

31. Li, Z; Lin, Z; Chen, L; Ba, H; Yang, Y; Wang, K; Wang, W; Qiang, Q; Li G. (2017): Draft genomic data of the Reindeer (Rangifer tarandus). GigaScience Database. http://dx.doi.org/100370

Figure legends

Figure 1. Male (above) and female (below) *Rangier tarandus* individuals, the only cervid species that both sexes are able to produce velvet antlers. Pictures courtesy of Yifeng Yang from the Institute of Special Animal and Plant Sciences, Chinese Academy of Agricultural Sciences.

Figure 2. Phylogenetic relationships of *Rangier tarandus* and four species based on four-fold degenerated sites. The blue numbers in the square brackets above the nodes are the 90% confidence interval of divergence time from the present. MYA, million years ago.

Tables

Table 1 Summary of genome assembly of *Rangier tarandus*

Type	Scaffold (bp)	Contig (bp)
Total number	58,765	117,102
Total length	2,832,785,815	2,732,476,387
N50 length	986,392	91,805
N90 length	151,297	17,480
Max length	4,664,725	770,474
GC content(%)	41.24	40.98

Click here to download Figure Reindeer_Figure1.docx

Figure 1. Male (above) and female (below) *Rangier tarandus* individuals, the only cervid species that both sexes are able to produce velvet antlers. Pictures courtesy of Yifeng Yang from the Institute of Special Animal and Plant Sciences, Chinese Academy of Agricultural Sciences.

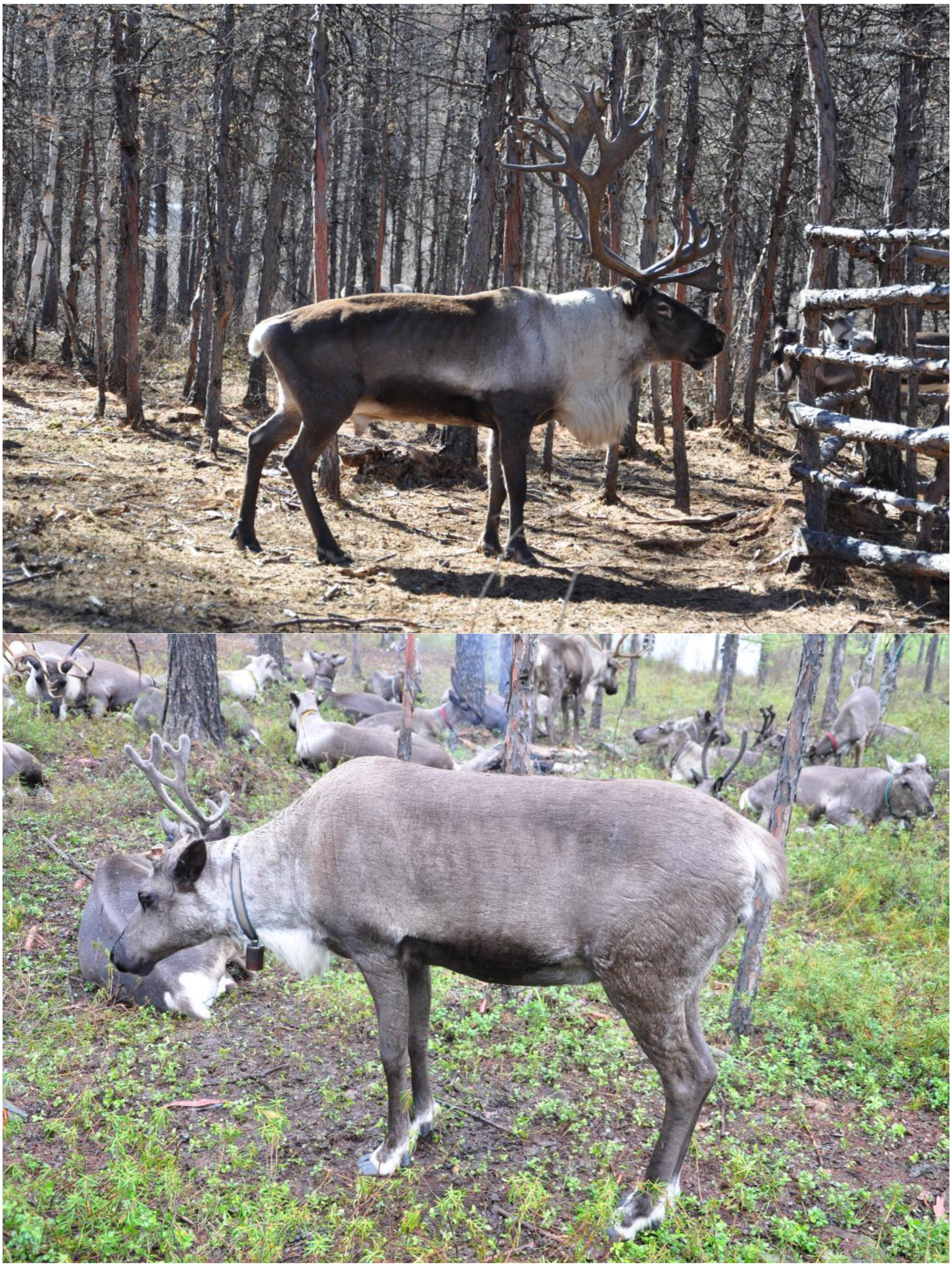

Figure 2

Click here to download Figure Figure1.pdf

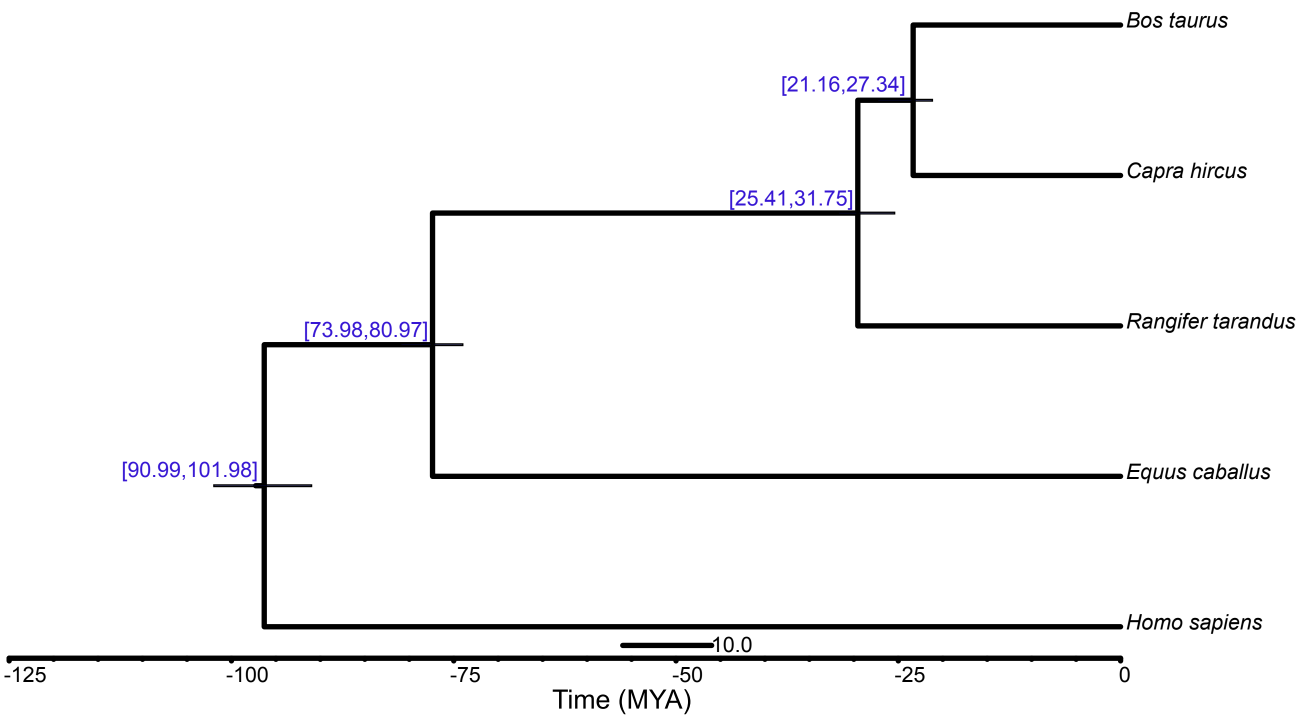

Click here to access/download
Supplementary Material
Supplementary tables_REVISED-1017.doc

Supplemental Figure 1

Click here to access/download
Supplementary Material
Figure S1.pdf

Supplemental Figure 2

Click here to access/download
Supplementary Material
Figure S2.pdf

Supplemental Figure 3

Click here to access/download
Supplementary Material
Fig.S3V7.pdf

Supplemental Figure 4

Click here to access/download
Supplementary Material
Figure S4.pdf

Supplemental Figure 5

Click here to access/download
Supplementary Material
Figure S5.pdf

Supplemental Figure 6

Click here to access/download
Supplementary Material
Figure S6.pdf

Supplemental Figure 7

Click here to access/download
Supplementary Material
Figure S7.pdf

 Click here to download Personal Cover Cover letter.docx

Dear editor of ***GigaScience***,

Thank you very much for your consideration on our manuscript (**ID GIGA-D-17-00152**) entitled "Draft genome of the Reindeer (*Rangifer tarandus*)" as a Data Note in ***GigaScience***.

We have carefully revised the manuscript according to the reviewers' comments and your instructions. The revised parts are marked with yellow. The point by point responses to the reviewer's comments are listed below, and **our responses are in bold and start with dashes "--".**

We hope that the revised manuscript has addressed all the concerns of reviewers and is now publishable

Yours sincerely,
Wen Wang, Ph.D
