# Supplementary material for: Draft genome of the reindeer (Rangifer tarandus)
Source: Gigascience. 2017 Nov 1;6(12):1–5. doi: 10.1093/gigascience/gix102 (PMC5726476; doi:10.1093/gigascience/gix102)
Supplement: Supplement Tables [file gix102_supplementary_tables_revised-1017.doc]

**Supplementary tables**

***Table S1 Summary of sequenced reads of*** Rangier tarandus

| Insert Size (bp) | Type | Bases sequenced (Gb) | Reads Length (bp) | Sequence Coverage (×)^a^ | Physical coverage (×)^a^ | Deposit number^b^ |
| --- | --- | --- | --- | --- | --- | --- |
| 200 | Paired-end | 78.82 | 150 | 28.61 | 19.07 | SRR5763132 |
| 250 | Paired-end | 74.96 | 150 | 27.20 | 22.66 | SRR5763133 |
| 350 | Paired-end | 78.62 | 150 | 28.53 | 33.28 | SRR5763130 |
| 400 | Paired-end | 91.43 | 150 | 33.18 | 44.22 | SRR5763131 |
| 450 | Paired-end | 105.47 | 150 | 38.28 | 57.39 | SRR5763128 |
| 3000 | Mate-paired | 81.26 | 150 | 29.49 | 293.48 | SRR5763129 |
| 6500 | Mate-paired | 64.53 | 150 | 23.42 | 498.86 | SRR5763126 |
| 11500 | Mate-paired | 72.35 | 150 | 26.25 | 1,002.05 | SRR5763127 |
| 16000 | Mate-paired | 75.80 | 150 | 27.51 | 1,463.27 | SRR5763125 |
| Total |  | 723.24 |  | 262.47 | 3,434.28 |  |

^a^ Sequence coverage is the average number of times a base is read, physical coverage is the average number of times a base spanned by paired or mate paired reads. ^b^ These data are deposited in SRA (SRR5763125-SRR5763133).

**Table S2 Summary of BUSCOa analysis**

| Types of BUSCOs | Count | Ratio |
| --- | --- | --- |
| Complete BUSCOs | 3,803 | 92.6% |
| Complete and single-copy BUSCOs | 3,687 | 89.8% |
| Complete and duplicated BUSCOs | 116 | 2.8% |
| Fragmented BUSCOs | 157 | 3.8% |
| Missing BUSCOs | 144 | 3.6% |

a. The lineage dataset is: mammalia_odb9 (Creation date: 2016-10-21, number of species: 50, number of BUSCOs: 4104)

**Table S3 Summary of breakpoints of *Rangier tarandus* and *Capra circus***

| Type of breakpoints | Numbers |
| --- | --- |
| Inter-chromosomal | 27,403 |
| Inversion | 3,313 |
| Translocation | 11,269 |
| Insertion | 71,745 |
| Deletion | 84,243 |
| Total | 197,973 |

**Table S4 Summary statics of interspersed** **repeat regions in *Rangier tarandus***

|  | Repbase TEs | | TE protiens | | De novo | | Combined TEs | |
| --- | --- | --- | --- | --- | --- | --- | --- | --- |
|  | Length (bp) | Percent (%) | Length (bp) | Percent (%) | Length (bp) | Percent (%) | Length (bp) | Percent (%) |
| DNA | 51,727,926 | 1.83 | 7,302,042 | 0.26 | 37,845,895 | 1.34 | 63,606,285 | 2.25 |
| LINE | 654,142,992 | 23.09 | 427,712,854 | 15.10 | 594,577,632 | 20.99 | 810,897,142 | 28.63 |
| SINE | 185,796,059 | 6.56 | 0 | 0.00 | 64,250,440 | 2.27 | 192,390,197 | 6.79 |
| LTR | 130,117,099 | 4.59 | 135,36,359 | 0.48 | 91,738,104 | 3.24 | 149,113,768 | 5.26 |
| Other | 0 | 0.00 | 0 | 0.00 | 0 | 0.00 | 0 | 0.00 |
| Unknown | 427,499 | 0.02 | 0 | 0.00 | 159,137,234 | 5.62 | 159,564,588 | 5.63 |
| Total | 1,011,576,167 | 35.71 | 448,373,780 | 15.83 | 912,174,882 | 32.20 | 1,109,868,568 | 39.18 |

**Table S5 General statistics of predicted protein-conding genes**

|  | Gene Set | Number | Average Transcript Length (bp) | Average CDS Length (bp) | Average Exon Number per Gene | Average Exon Length(bp) | Average Intron Length (bp) |
| --- | --- | --- | --- | --- | --- | --- | --- |
| De novo | AUGUSTUS | 24,814 | 39,409.58 | 1,306.89 | 7.62 | 171.57 | 5,758.21 |
|  | GENSCAN | 37,987 | 33,288.39 | 1,171.25 | 6.99 | 167.55 | 5,361.35 |
|  | GLIMMERHMM | 24,300 | 9,918.45 | 846.62 | 4.54 | 186.43 | 2,561.74 |
|  | SNAP | 86,156 | 35,898.49 | 756.65 | 6.36 | 118.88 | 6,550.44 |
| Homolog | *H.sapiens* | 32,440 | 17,507.2 | 1,147.99 | 6.20 | 185.25 | 3,147.83 |
|  | *B.taurus* | 29,167 | 18,212.38 | 1,248.30 | 6.75 | 185.07 | 2,952.74 |
|  | *O.aries* | 30,742 | 17,886.14 | 1,222.49 | 6.62 | 184.68 | 2,965.27 |
| Final | -- | 21,555 | 29,663.02 | 1,440.48 | 8.19 | 175.98 | 3,927.62 |

**Table S6 Summary statistics of gene function annotationa**

| Annotation type | Numbers | Percent(%) |
| --- | --- | --- |
| InterPro | 17,513 | 81.25 |
| GO | 14,138 | 65.595 |
| KEGG | 5,240 | 24.31 |
| Swissprot | 18,536 | 85.99 |
| TrEMBL | 18,892 | 87.65 |
| Annotated | 19,004 | 88.17 |
| Unanotated | 2,551 | 11.83 |

a Total gene numbers: 21,555.

**TableS7 The distribution of single nucleotide variant (SNV)** in the reindeer genome.

| Type (alphabetical order) | Count | Percent |
| --- | --- | --- |
| 5_prime_UTR_variant | 5 | 0.00% |
| Conservative inframe deletion | 52 | 0.00% |
| Conservative inframe insertion | 48 | 0.00% |
| Disruptive inframe deletion | 26 | 0.00% |
| Disruptive inframe insertion | 16 | 0.00% |
| Downstream gene variant | 104,268 | 3.11% |
| Feature elongation | 1 | 0.00% |
| Frameshift variant | 168 | 0.01% |
| Intergenic region | 2,488,040 | 74.20% |
| Intron variant | 632,370 | 18.86% |
| Missense variant | 9,028 | 0.27% |
| Non coding transcript variant | 3 | 0.00% |
| Splice acceptor variant | 47 | 0.00% |
| Splice donor variant | 57 | 0.00% |
| Splice region variant | 1,910 | 0.06% |
| Start lost | 39 | 0.00% |
| Stop gained | 165 | 0.01% |
| Stop lost | 35 | 0.00% |
| Stop retained variant | 4 | 0.00% |
| Synonymous variant | 10,826 | 0.32% |
| Upstream gene variant | 106,239 | 3.17% |
| total | 3,353,347 | 100.00% |

**TableS8 Summary of ncRNA annotation**

| Type |  | Copy (w) | Average length (bp) | Total length (bp) | Percent (%) |
| --- | --- | --- | --- | --- | --- |
| miRNA |  | 547 | 92.9049 | 50,819 | 0.0017 |
| tRNA |  | 863 | 73.1483 | 63,127 | 0.0022 |
| rRNA | rRNA | 159 | 91.4088 | 14,534 | 0.0005 |
|  | 18S | 16 | 91.8125 | 1,469 | 0.0000 |
|  | 28S | 36 | 140.3611 | 5,053 | 0.0002 |
|  | 5.8S | 2 | 130.5000 | 261 | 0.0000 |
|  | 5S | 105 | 73.8191 | 7,751 | 0.0003 |
| snRNA | snRNA | 1,339 | 119.7214 | 160,307 | 0.0056 |
|  | CD-box | 102 | 130.8235 | 13,344 | 0.0004 |
|  | HACA-box | 267 | 137.9588 | 36,835 | 0.0013 |
|  | splicing | 951 | 112.6330 | 107,114 | 0.0037 |
