# Supplementary figures and images for: Draft genome of the reindeer (Rangifer tarandus)
Source: Gigascience. 2017 Nov 1;6(12):1–5. doi: 10.1093/gigascience/gix102 (PMC5726476; doi:10.1093/gigascience/gix102)

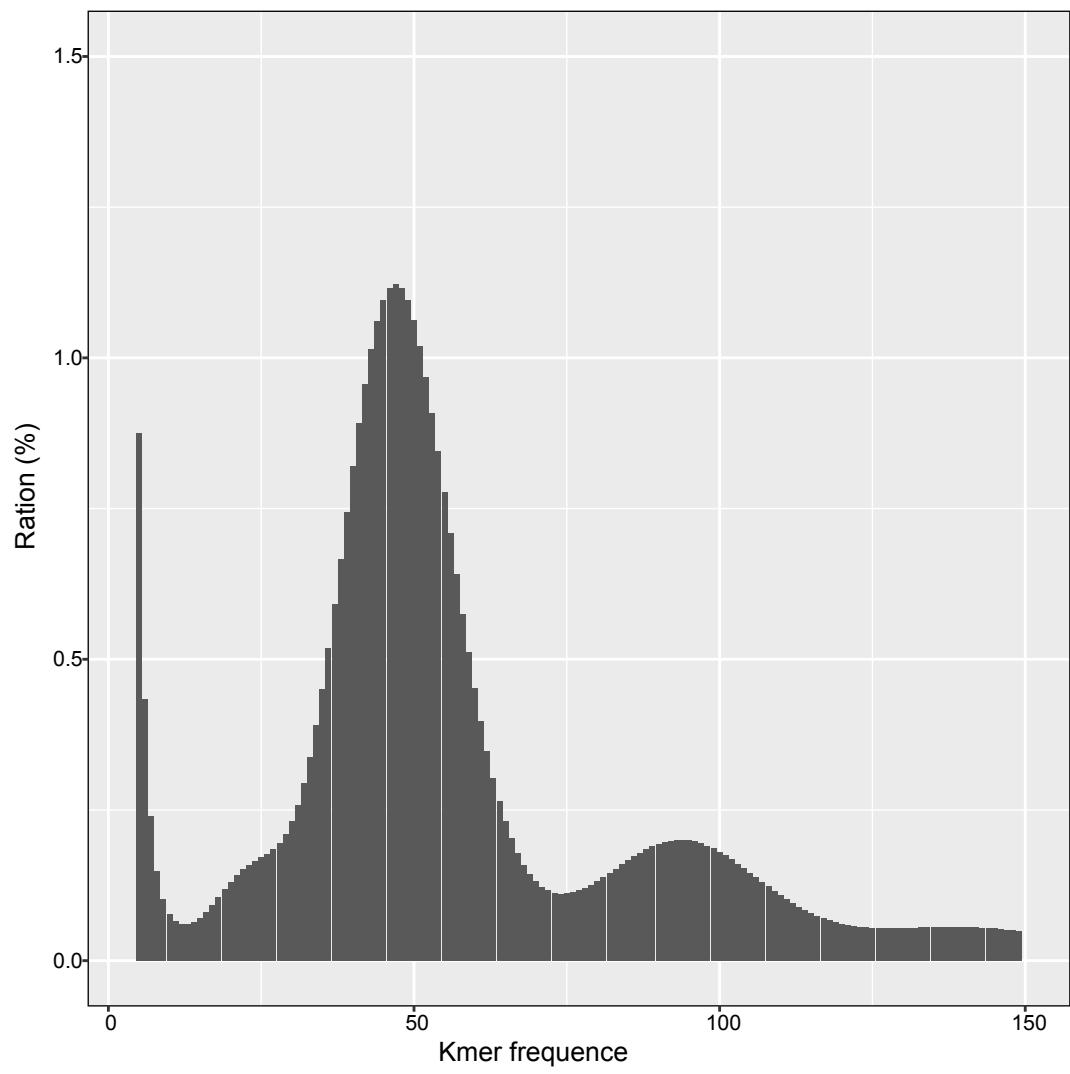

Supplement: Supplement Figure S1 [file gix102_figure_s1.pdf]

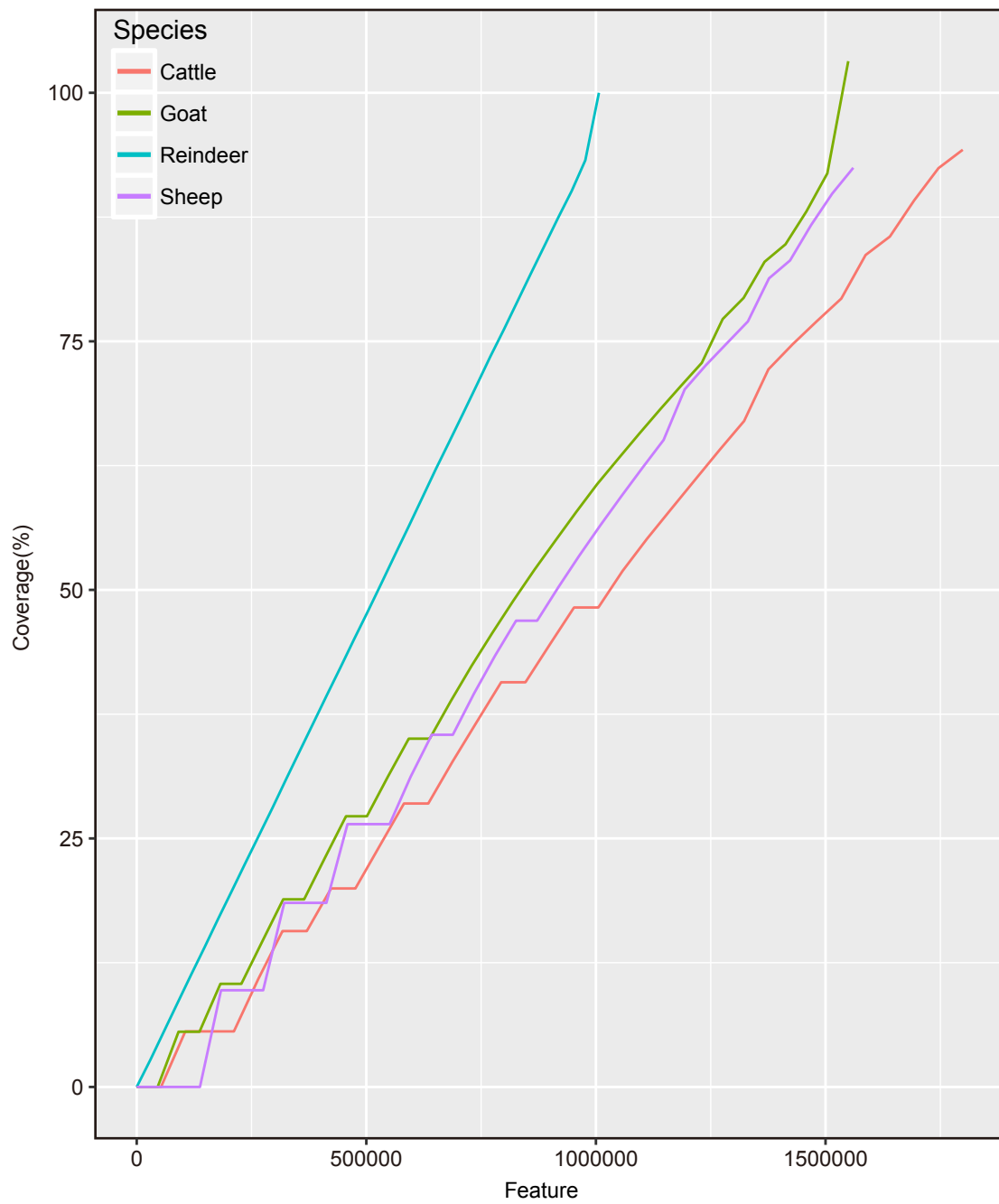

Supplement: Supplement Figure S2 [file gix102_figure_s2.pdf]

Scaffolds of *Rangier tarandus* genome

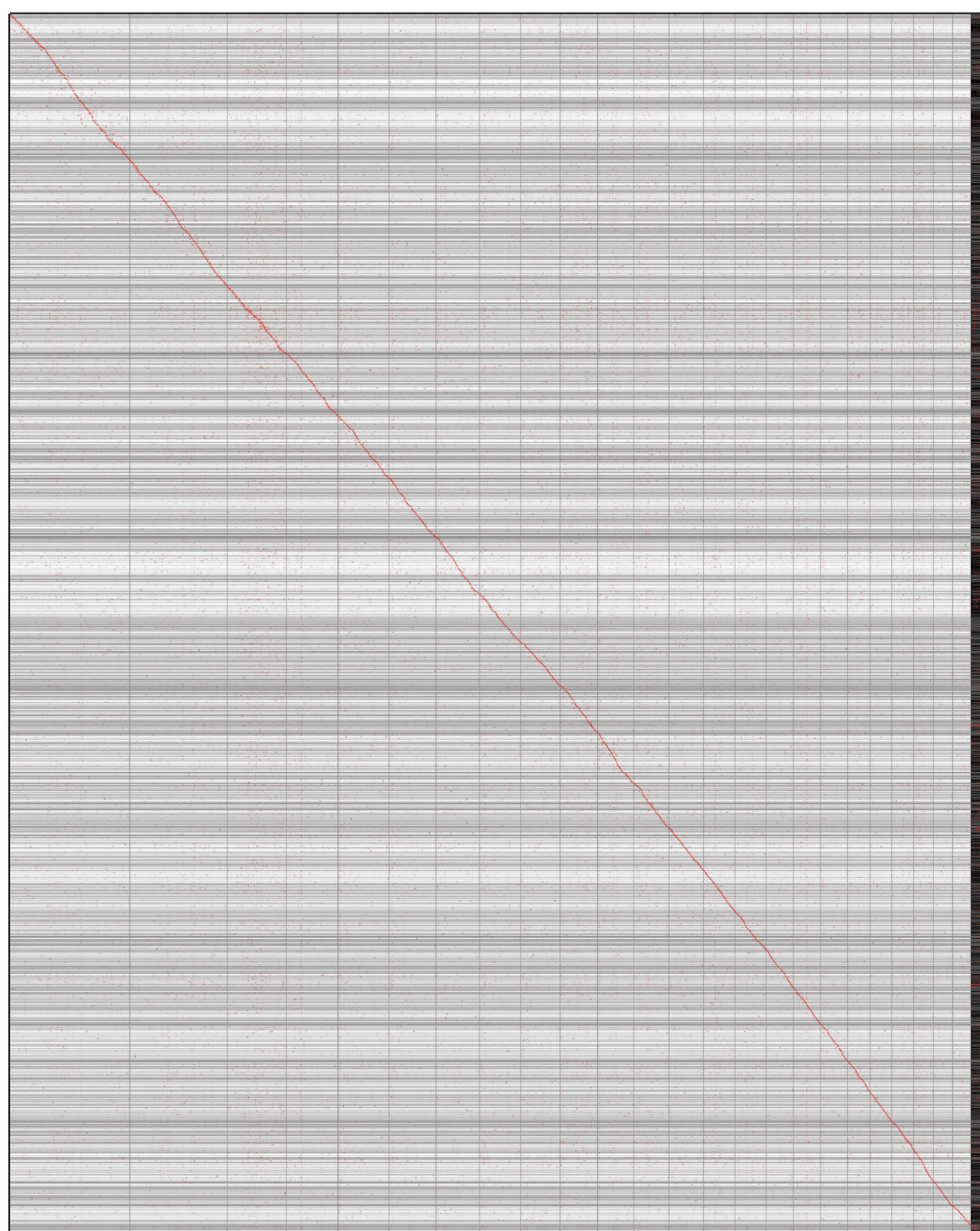

Chromosomes of *Capra hircus* genome

Supplement: Supplement Figure S3 [file gix102_figure_s3.pdf]

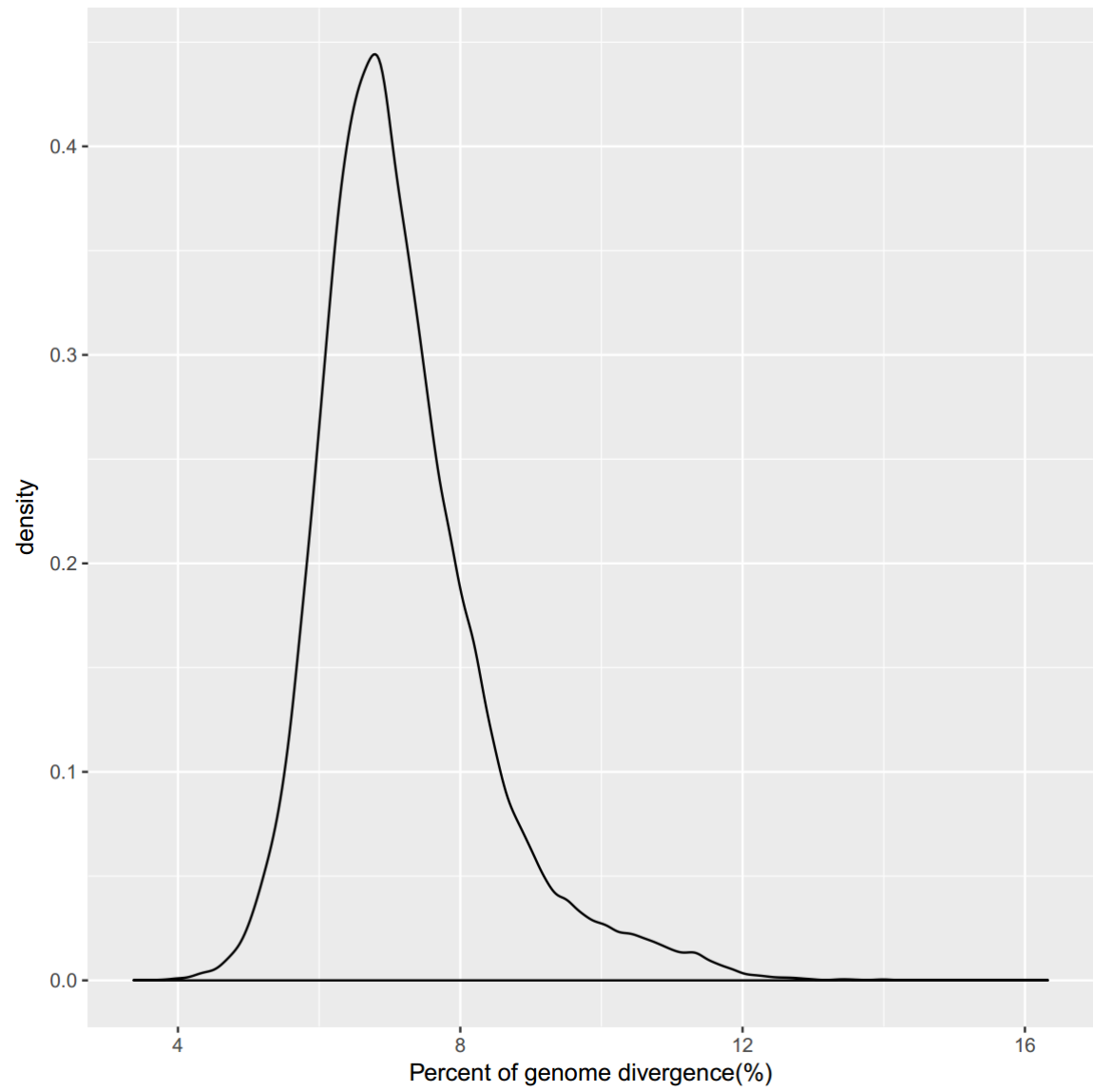

Supplement: Supplement Figure S4 [file gix102_figure_s4.pdf]

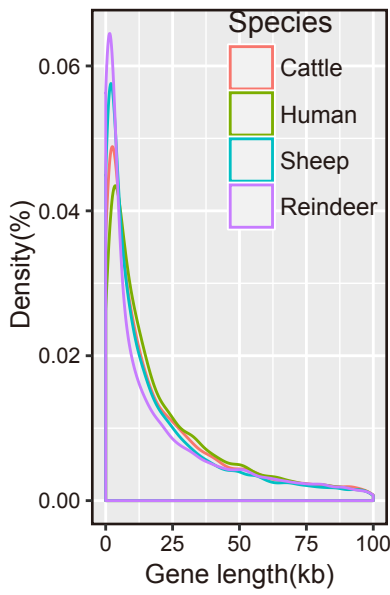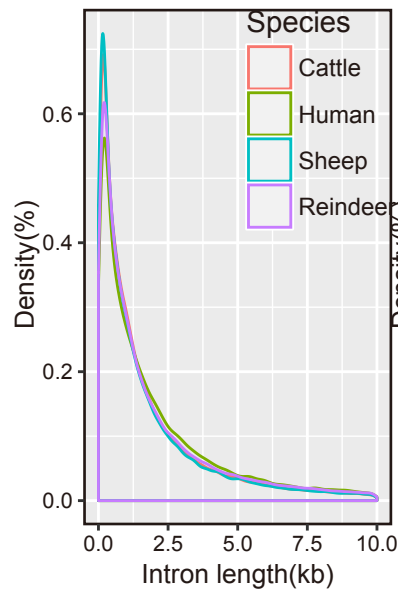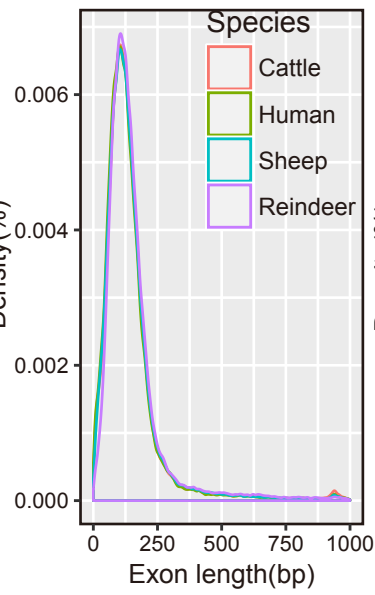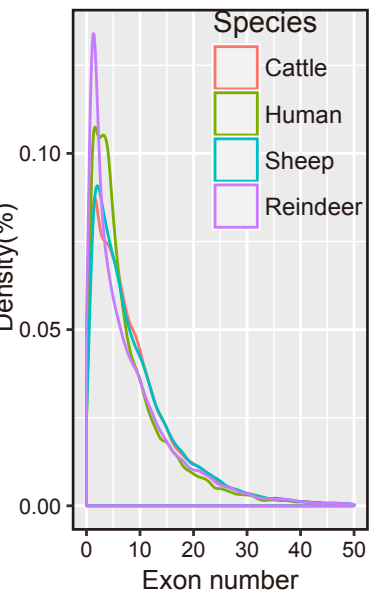

Supplement: Supplement Figure S5 [file gix102_figure_s5.pdf]

# Reindeer GO Annotation

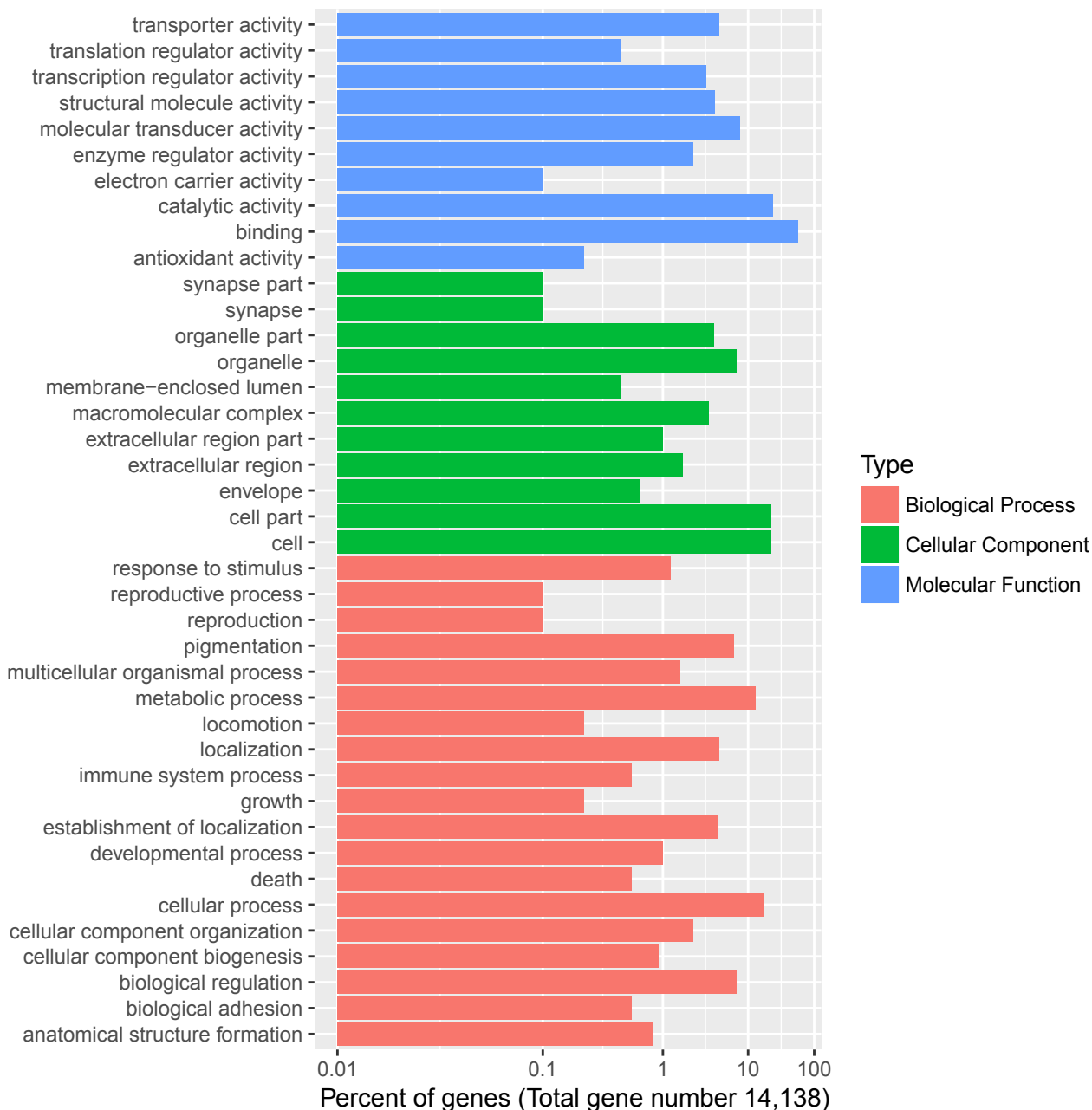

Supplement: Supplement Figure S6 [file gix102_figure_s6.pdf]

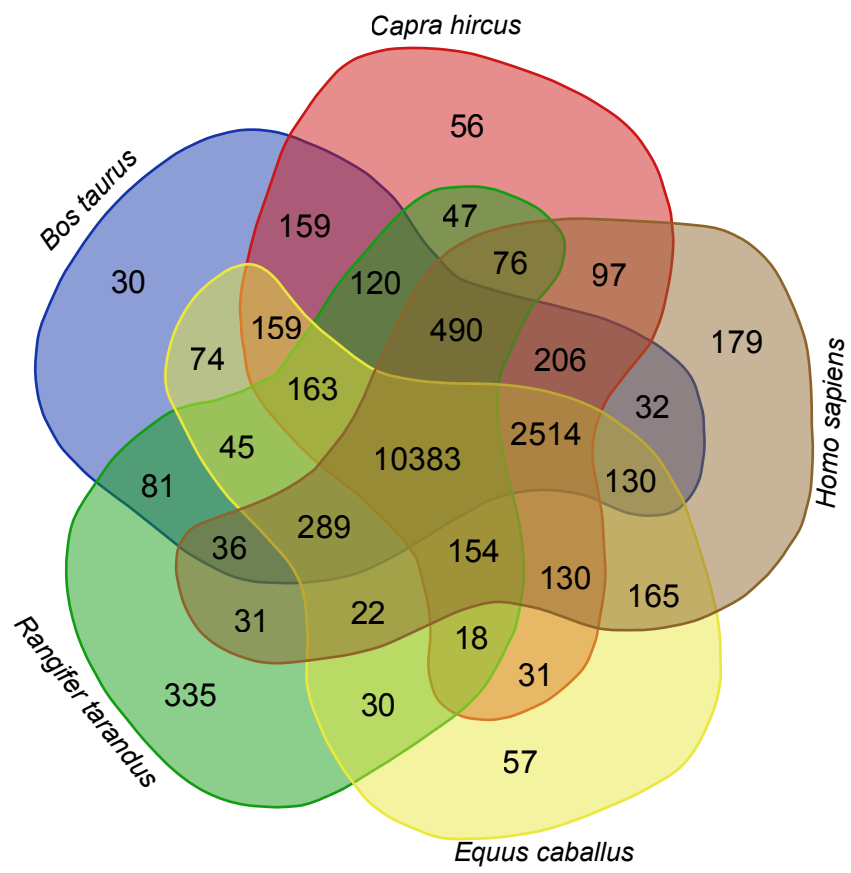

Supplement: Supplement Figure S7 [file gix102_figure_s7.pdf]
