# Supplementary material for: Draft genome of the reindeer (Rangifer tarandus)
Source: Gigascience. 2017 Nov 1;6(12):1–5. doi: 10.1093/gigascience/gix102 (PMC5726476; doi:10.1093/gigascience/gix102)
Supplement: gix102_Response-to-Reviewer-Comments_Original-Submission.pdf [file gix102_response-to-reviewer-comments_original-submission.pdf]

Author's Response To Reviewer Comments

We have carefully revised the manuscript according to the reviewers' comments. The point by point responses to the reviewer's comments are listed below, and our responses are in bold and start with dashes "--".
Reviewer reports:
Reviewer #1
In this Data Note the authors describe the first genome assembly for the reindeer. I am impressed by the amount and variety of analyses undertaken to demonstrate the quality of the assembly. That said, I think there are places in the manuscript where the methods could be more fully explained, and broader context given to the results. Specific comments are below
--We greatly thank the reviewer to provide us these positive comments to improve the manuscript, especially in the method and result sections.

Line 20: could be fair to mention that the amount of usable sequence was actually 615 Gb (line 66)
--We change this sentence to "We obtained 615 Gb (Gigabase) usable sequences by filtering the low quality reads of the raw data generated from Illumina Hiseq 4000 platform" in lines 21-22.

Lines 42-45: these two sentences should be re-worded for clarity.
--Thanks for your suggestion. In these sentences, we want to address the importance of antlers and the interesting biology of reindeer, which is the only species that females grow antler in the Cervidae. We rewrite these sentences as "Antlers are the defining characteristic of male cervids, belonging to the secondary sexual appendage, which shed and regrow in each year throughout an animal's life. Interestingly, reindeer is the only species that females regularly grow antlers in cervids." in lines 43-46.

Line 49: replace "special" with "this"
-- Changed as suggested.

Table S1: what is the difference between sequence and physical converge?
-- Sequence coverage is the average number of times a base is read, physical coverage is the average number of times a base spanned by paired or mate paired reads. We have added the explanation in the note of the table.

Lines 69-71: a fuller explanation of the k-mer analysis would be useful. Also, I noted that the distribution in Figure S1 is bimodal. Is this expected? Is it a problem for the analysis? Finally, why not use the traditional c-value estimate of genome size, or at least provide a comparison of the two estimates?
-- We have now fully explained the k-mer analysis in lines 70-73 on page 5. The bimodal is common in this kind of analysis. A k-mer is related to an artificial sequence division of K nucleotides extracted iteratively from sequencing reads. We defined the k-mer length as 17 bp; thus, a L bp-long clean sequence would include (L-17 + 1) k-mers. The frequency of each k-mer can be calculated from the genome sequence reads. Typically, k-mer frequencies plotted against the sequence depth gradient follows a Poisson distribution in any given dataset. The k-mer method is regularly used to estimate genome size and heterozogosity in genome projects, and C

value method is only used in some less studied taxa with unknown genome size range while the reindeer is one of mammals, whose genome sizes are relatively stable.

Lines 87-89: it is stated that the accumulation curves in Figure S2 are similar, but to me it looks like the slope for the reindeer is much steeper and more linear than the other genomes. Are they statistically the same? If the reindeer one is different why might that be?
-- The horizontal axis represents the error rate and the vertical axis represents the coverage. The error rate of the reindeer is the lowest at the same coverage, indicating that the high quality assembly of reindeer genome. We have added the explanation in the legend of Figure S2.

Lines 89-96: why was the goat genome chosen for syteny analyses? Is not the cow genome more complete?
-- The goat genome is generated by the third-generation sequencing technology recently with much longer contigs and higher accuracy compared to other ruminants.

Figure S3: please expand the figure legend so that it contains more information as to what is being shown.
-- Thanks for the suggestion. We have improved Figure S3 and added more explanation: The horizontal and vertical axis represents the chromosomes of goat (Capra hircus) and the scaffolds of reindeer (Rangier tarandus), respectively. Those red dots indicate the collinear regions of the two genomes.

Table S4: indicate where % corresponds to % of the genome versus % of elements found.
-- The % in Table S4 indicates that the percentage of repeat regions in reindeer genome. Moreover, we checked the results again and corrected some mistakes which are now marked in yellow.

I would suggest moving the reference to Table S6 from the end of Line 128 to the end of the sentence on Line 127. As it stands now when I went to look at the data I was expecting to see a summary of the functions annotated, not a comparison of how the different software's did. That said, a table summarizing the functions annotated would also be interesting.
-- Thanks for the suggestions. As suggested we have added a Gene ontology annotation to indicate distribution of gene functions in Figure S6.

Lines 130-131: state how many variants were found.
-- We added a sentence in lines 135-136 on page 8 "Finally, a total of 3,353,347 SNVs were found in the genome of reindeer (Table S7)."

Lines 151-153: is this divergence time in line with previous estimates? Please provide citations.
-- The estimated divergence time is consistent with the published results (Ref 1 and 2 listed below). We cited these papers in our manuscript in lines 157-158 of pages 9. "This is consistent with the previous findings from both fossil record and molecular phylogeny analysis (Ref 1 and 2). "
Ref 1. dos Reis M, Inoue J, Hasegawa M, Asher RJ, Donoghue PCJ and Yang Z. Phylogenomic datasets provide both precision and accuracy in estimating the timescale of placental mammal phylogeny. Proceedings of the Royal Society B: Biological Sciences. 2012;279 1742:3491-500.

Ref 2. Bibi F. A multi-calibrated mitochondrial phylogeny of extant Bovidae (Artiodactyla, Ruminantia) and the importance of the fossil record to systematics. BMC Evol Biol. 2013;13 1:166.

Reviewer #2
This is an extremely useful paper to those that are interested in farmed ruminants especially cervids. There are some minor typographical errors which are described below and several minor queries whose answers would improve the text. I checked through the ftp site and the annotation information looks very good and useful. I did not however, download and check each file.
--Thank you very much for your positive comments on this work.

Comments
Major
I am rather confused as nowhere in the text is it described how the assembly scaffolds were ordered and aligned into chromosomes and or genome order. This is important as a number of analyses depend on this aspect. For example the genome comparison with goat (Figure S3V5) and I note the axes of this figure are also cryptically not annotated with either bp or chromosome numbers. I suspect that this did not happen except via homology comparison with another species (sheep or cattle, maybe goat?). Why raise this point? Well to me a high quality assembly actually rests on the scaffolds being ordered and orientated based on data like Hi-C, optical mapping, linkage mapping, LD mapping, or radiation hybrids of which there is no mention. This aspect needs to be clarified and described and commented on.
--We are sorry for any unclearness in the description of genome assembly. Indeed, this work didn’t assemble the reindeer genome to the chromosomal level, but only to the draft (regular scaffold) level. We actually aligned the reindeer scaffolds to the goat genome which was assembled to the chromosomal level (Ref 3 listed below) to evaluate the quality of our draft assembly. So chromosome information in Figure S3 etc refers to those of goat rather than the reindeer. Usually a high quality draft genome assembly is enough for most biological analyses. If the reindeer chromosomal level information is needed in future studies, one indeed has to use Hi-C, optical mapping, or genetic maps generated with methods like radiation hybrids, and even Hi-C and optical mapping usually can only get longer scaffolds rather than complete chromosomes. We have explained more in the Figure S3 legend avoid ambiguity.

Ref 3. Bickhart DM, Rosen BD, Koren S, Sayre BL, Hastie AR, Chan S, et al. Single-molecule sequencing and chromatin conformation capture enable de novo reference assembly of the domestic goat genome. Nat Genet. 2017;49 4:643-50.

Minor * line 19 "species" rather than "specie"
-- Corrected as suggested.

* line 58 The DNA extraction method (and reference) are not described. It is also impossible to call blood frozen and then presumably thawed "fresh blood". This makes me suspect this aspect is unclear to the authors.
-- Thank the reviewer to correct this statement. The DNA was extracted from the thawed blood. We rewrite this sentence to "Genomic DNA was extracted from the sample thawed from frozen

blood using the DNeasy Blood & Tissue Kit (QIAGEN, Valencia, CA, USA) according to the manufacturer’s instructions." in lines 58-60 on page 5.

* Table s1 needs web address for the deposit numbers.
-- We have added the deposit numbers in the Table S1 and their web link.

* line 153 I suspect figure 1 = figure S7? Figure 1 the precision in the estimated divergence times is excessive and the legend should be altered to make clear it is a range.
-- Thank for pointing out this. We have now changed “Figure1” to “Figure S7” in line 157 of page 9. And we used a range rather than a concrete number for the time divergence. We have rewritten the legend of Figure 1 making it clearer.

* line 168 "he" should be "the".
-- Sorry for the typo, we have corrected it.

* line 172 "libraries" should be "library".
-- Corrected as suggested.
