# Supplementary material for: Draft genome of the reindeer (Rangifer tarandus)
Source: Gigascience. 2017 Nov 1;6(12):1–5. doi: 10.1093/gigascience/gix102 (PMC5726476; doi:10.1093/gigascience/gix102)
Supplement: gix102_Reviewer-1-Report-(Original-Submission).pdf [file gix102_reviewer-1-report-(original-submission).pdf]

Reviewer Report

Title: Draft genome of the Reindeer (Rangifer tarandus)

Version: Original Submission **Date:** 7/16/2017

Reviewer name: Joshua Moses Miller

Reviewer Comments to Author:

In this Data Note the authors describe the first genome assembly for the reindeer. I am impressed by the amount and variety of analyses undertaken to demonstrate the quality of the assembly. That said, I think there are places in the manuscript where the methods could be more fully explained, and broader context given to the results. Specific comments are belowLine 20: could be fair to mention that the amount of usable sequence was actually 615 Gb (line 66)Lines 42-45: these two sentences should be re-worded for clarity.Line 49: replace "special" with "this"Table S1: what is the difference between sequence and physical converge?Lines 69-71: a fuller explanation of the k-mer analysis would be useful. Also, I noted that the distribution in Figure S1 is bimodal. Is this expected? Is it a problem for the analysis? Finally, why not use the traditional c-value estimate of genome size, or at least provide a comparison of the two estimates?Lines 87-89: it is stated that the accumulation curves in Figure S2 are similar, but to me it looks like the slope for the reindeer is much steeper and more linear than the other genomes. Are they statistically the same? If the reindeer one is different why might that be?Lines 89-96: why was the goat genome chosen for syteny analyses? Is not the cow genome more complete?Figure S3: please expand the figure legend so that it contains more information as to what is being shown.Table S4: indicate where % corresponds to % of the genome versus % of elements found.I would suggest moving the reference to Table S6 from the end of Line 128 to the end of the sentence on Line 127. As it stands now when I went to look at the data I was expecting to see a summary of the functions annotated, not a comparison of how the different software's did. That said, a table summarizing the functions annotated would also be interesting.Lines 130-131: state how many variants were found.Lines 151-153: is this divergence time in line with previous estimates? Please provide citations.

Level of Interest

Please indicate how interesting you found the manuscript: An article whose findings are important to those with closely related research interests

Quality of Written English

Please indicate the quality of language in the manuscript: Acceptable

Declaration of Competing Interests

Please complete a declaration of competing interests, considering the following questions:

- Have you in the past five years received reimbursements, fees, funding, or salary from an organisation that may in any way gain or lose financially from the publication of this manuscript, either now or in the future?
- Do you hold any stocks or shares in an organisation that may in any way gain or lose financially from the publication of this manuscript, either now or in the future?
- Do you hold or are you currently applying for any patents relating to the content of the manuscript?
- Have you received reimbursements, fees, funding, or salary from an organization that holds or has applied for patents relating to the content of the manuscript?
- Do you have any other financial competing interests?
- Do you have any non-financial competing interests in relation to this paper?

If you can answer no to all of the above, write 'I declare that I have no competing interests' below. If your reply is yes to any, please give details below.

I declare that I have no competing interests.

I agree to the open peer review policy of the journal. I understand that my name will be included on my report to the authors and, if the manuscript is accepted for publication, my named report including any attachments I upload will be posted on the website along with the authors' responses. I agree for my report to be made available under an Open Access Creative Commons CC-BY license (http://creativecommons.org/licenses/by/4.0/). I understand that any comments which I do not wish to be included in my named report can be included as confidential comments to the editors, which will not be published.

I agree to the open peer review policy of the journal
