# Supplementary material for: Draft genome of the reindeer (Rangifer tarandus)
Source: Gigascience. 2017 Nov 1;6(12):1–5. doi: 10.1093/gigascience/gix102 (PMC5726476; doi:10.1093/gigascience/gix102)
Supplement: gix102_Reviewer-2-Report-(Original-Submission).pdf [file gix102_reviewer-2-report-(original-submission).pdf]

Reviewer Report

Title: Draft genome of the Reindeer (Rangifer tarandus)

Version: Original Submission **Date:** 9/16/2017

Reviewer name: John McEwan

Reviewer Comments to Author:

This is an extremely useful paper to those that are interested in farmed ruminants especially cervids. There are some minor typographical errors which are described below and several minor queries whose answers would improve the text. I checked through the ftp site and the annotation information looks very good and useful. I did not however, download and check each file.CommentsMajorI am rather confused as nowhere in the text is it described how the assembly scaffolds were ordered and aligned into chromosomes and or genome order. This is important as a number of analyses depend on this aspect. For example the genome comparison with goat (Figure S3V5) and I note the axes of this figure are also cryptically not annotated with either bp or chromosome numbers. I suspect that this did not happen except via homology comparison with another species (sheep or cattle, maybe goat?). Why raise this point? Well to me a high quality assembly actually rests on the scaffolds being ordered and orientated based on data like Hi-C, optical mapping, linkage mapping, LD mapping, or radiation hybrids of which there is no mention. This aspect needs to be clarified and described and commented on.minor* line 19 "species" rather than "specie"* line 58 The DNA extraction method (and reference) are not described. It is also impossible to call blood frozen and then presumably thawed "fresh blood". This makes me suspect this aspect is unclear to the authors.* Table s1 needs web address for the deposit numbers* line 153 I suspect figure 1 = figure S7? Figure 1 the precision in the estimated divergence times is excessive and the legend should be altered to make clear it is a range.* line 168 "he" should be "the"* line 172 "libraries" should be "library"

Level of Interest

Please indicate how interesting you found the manuscript: An article of importance in its field

Quality of Written English

Please indicate the quality of language in the manuscript: Needs some language corrections before being published

Declaration of Competing Interests

Please complete a declaration of competing interests, considering the following questions:

- Have you in the past five years received reimbursements, fees, funding, or salary from an organisation that may in any way gain or lose financially from the publication of this manuscript, either now or in the future?
- Do you hold any stocks or shares in an organisation that may in any way gain or lose financially from the publication of this manuscript, either now or in the future?
- Do you hold or are you currently applying for any patents relating to the content of the manuscript?
- Have you received reimbursements, fees, funding, or salary from an organization that holds or has applied for patents relating to the content of the manuscript?
- Do you have any other financial competing interests?
- Do you have any non-financial competing interests in relation to this paper?

If you can answer no to all of the above, write 'I declare that I have no competing interests' below. If your reply is yes to any, please give details below.

I declare that i have no competing interests

I agree to the open peer review policy of the journal. I understand that my name will be included on my report to the authors and, if the manuscript is accepted for publication, my named report including any attachments I upload will be posted on the website along with the authors' responses. I agree for my report to be made available under an Open Access Creative Commons CC-BY license (http://creativecommons.org/licenses/by/4.0/). I understand that any comments which I do not wish to be included in my named report can be included as confidential comments to the editors, which will not be published.

I agree to the open peer review policy of the journal
